# Adult Age Threshold Estimation Using Radiographic Evaluation of Wrist–Hand Skeletal Maturation: A Systematic Review and Meta-Analysis

**DOI:** 10.3390/diagnostics16132093

**Published:** 2026-07-03

**Authors:** Ilenia Bianchi, Martina Focardi, Andrea Costantino, Beatrice Defraia, Vilma Pinchi

**Affiliations:** 1 Laboratory of Personal Identification and Forensic Morphology, Department of Health Sciences, University of Florence, 50139 Florence, Italy; ilenia.bianchi@unifi.it (I.B.); vilma.pinchi@unifi.it (V.P.); 2 Multidisciplinary Research Laboratory in Forensic Sciences (CRIME-LAB), Department of Health Sciences, University of Florence, 50139 Florence, Italy; 3 Forensic Pathology Unit, AOU Careggi, 50134 Florence, Italybeatrice.defraia@unifi.it (B.D.)

**Keywords:** adult age estimation, skeletal maturation, Greulich-Pyle, Tanner-Whitehouse, wrist-hand X-rays, meta-analysis

## Abstract

**Background:** Wrist-hand radiographic skeletal maturation methods are widely used for forensic age estimation of living individuals, remaining among the most widely accepted imaging modalities in forensic practice despite limited evidence supporting their use at the legally relevant 18-year threshold. This systematic review aims to provide a quantitative synthesis of the widely recognized but previously unsynthesized body of evidence regarding their diagnostic accuracy (sensitivity, specificity, AUC-curve, mean absolute error-MAE) for forensic adult age estimation. **Methods:** Six databases (Scopus, PubMed, Google Scholar, Embase, Cochrane, Clarivate) between 1980 and 2026 were searched. Studies evaluating wrist-hand skeletal maturation for forensic age estimation in individuals ≥16 years with verified chronological age were included. Two reviewers independently screened studies and extracted data. Quality was assessed using QUADAS-2. DerSimonian-Laird random-effects model estimated the sensitivity and specificity of the wrist-hand bones maturation at the age of 18 years, and pooled MAE. GRADE assessed the certainty of evidence. **Results:** From 747 records, 23 studies (11,425 participants, 15 countries, 2003–2025) were included. Pooled MAE was 0. 537 years (95% CI: 0.387–0.686; 95% PI: 0.33–0.79 years), but obtained from 3 Italian studies conducted on clinical populations, and all characterized by high risk of bias. At the 18-year threshold, pooled sensitivity was 69.5% (95% CI: 61.6–77.3%; 95% PI: 35.3–100%) and specificity 85.56% (95% CI: 83.53–87.60%; 95% PI: 77.2–93.9%). Nevertheless, the heterogeneity was extreme (I^2^ > 98%) for all diagnostic measures, and calculated prediction intervals confirm that individual study results are expected to vary widely. QUADAS-2 assessment revealed 82.6% of studies with high/unclear patient selection bias. GRADE-certainty was VERY LOW for both outcomes due to serious risk of bias, very serious inconsistency, and serious indirectness. **Conclusions:** Wrist-hand skeletal maturation shows low accuracy and high rates of misclassification for age estimation at the 18-year threshold. The wide prediction intervals (sensitivity 35–100%, specificity 77–94%, MAE 0.33–0.79 years) indicate that performance in a new study population may differ substantially from the pooled estimates. Very low certainty evidence, extreme heterogeneity, and substantial methodological limitations preclude confident application. Results should be interpreted with caution in forensic contexts, preferably combining multiple age indicators, in particular dental evidence.

## 1. Introduction

The increasing prevalence of unaccompanied minors seeking asylum, criminal proceedings necessitating age verification, and child protection cases [1,2] has significant social and forensic implications for biological age estimation practice, particularly concerning legal age thresholds (16, 18, and 21 years) when documentary evidence of age is unavailable [3,4]. The determination of whether an individual has attained legal majority (typically 18 years in most jurisdictions) represents a critical forensic practice, as misclassification of a minor as an adult carries serious ramifications, including inappropriate detention, denial of child protection services, and exposure to adult criminal justice systems [5,6]. This high-stakes medicolegal context necessitates rigorous evaluation of the accuracy, reliability, and inherent limitations of the available age estimation methods.

Skeletal maturation assessment remains widely applied for adult threshold determination, particularly through evaluation of wrist-hand bone maturation stages using atlas-based and scoring systems [7,8,9,10,11,12,13,14,15]. Certain approaches focus specifically on the timing of epiphyseal fusion at the distal radius, ulna, and metacarpals, processes that continue into late adolescence and early adulthood [15]. Recent technological advances have introduced machine learning and deep learning algorithms (AI/ML) for automated skeletal age assessment [16,17]. Commercial systems such as BoneXpert have gained acceptance in clinical settings [18]. However, forensic validation remains limited. Despite decades of research and widespread forensic application, critical knowledge gaps persist regarding the diagnostic accuracy of wrist-hand maturation assessment for correctly classifying individuals relative to the 18-year age threshold. Several studies have revealed significant inconsistencies in the precision and accuracy of skeletal methods based on wrist-hand maturation for age estimation in the 17–19 year age cohort [19,20,21,22].

In a forensic context, wrist-hand skeletal maturation methods are widely recognized to have limited accuracy for age estimation around the legally relevant threshold of 18 years; they remain more acceptable in several countries than radiographic examinations of other anatomical regions, particularly dental methods based on third molar assessment, with the latter having consistently demonstrated superior accuracy for estimating age in individuals older than 16 years. This is because dental radiographs are not uniformly accepted for forensic purposes in several jurisdictions owing to ethical, legal, cultural, or procedural constraints [19,23]. Consequently, hand-wrist radiography remains one of the most frequently applied and readily accessible imaging modalities in forensic age assessment practice in many countries. The methodological quality of primary studies on skeletal methods varies considerably, with concerns including patient selection bias, absence of blinding procedures, inadequate reference standards, and selective outcome reporting [19,24]. Furthermore, emerging AI-based methods require rigorous validation against established approaches to determine whether they offer substantive improvements in accuracy, reliability, and applicability within forensic contexts [25]. The comparative performance of different methods across diverse populations and age ranges remains inadequately characterized [21,22].

For this reason, there is still a clear need to systematically evaluate whether reliable evidence exists for the use of wrist-hand skeletal maturation methods specifically at the legally critical threshold of 18 years, and to assess the overall strength and quality of that evidence.

To the best of our knowledge, no comprehensive systematic review with meta-analysis has synthesized the totality of evidence regarding wrist-hand skeletal maturation for forensic age estimation at the 18-year threshold. Consequently, critical questions remain unresolved: What is the certainty of evidence supporting current forensic practice for adulthood threshold determination? What are the implications for forensic practitioners, legal decision-makers, and policymakers?

The primary objective of this systematic review and meta-analysis is to provide a quantitative synthesis of a widely recognized but previously non-systematically evaluated body of evidence, thereby clarifying the actual diagnostic performance, heterogeneity, and evidentiary limitations of these methods in forensic practice. This systematic review comprehensively evaluates the diagnostic accuracy and reliability (sensitivity, specificity, area under the curve-AUC, mean absolute error-MAE) of wrist-hand radiographic skeletal maturation methods (Greulich-Pyle-GP, Tanner-Whitehouse-TW, AI/ML approaches) for forensic age estimation in determining whether individuals have reached legal age thresholds, specifically the 18-year threshold.

## 2. Materials and Methods

### 2.1. Review Protocol and Research Question

This systematic review was conducted following the Preferred Reporting Items for Systematic Reviews and Meta-Analyses (PRISMA) 2020 guidelines [26] and the PRISMA extension for Diagnostic Test Accuracy (PRISMA-DTA) [27]. A completed search strategy and PRISMA 2020 checklist are provided in Supplementary Materials—Supplementary S1–S3.

The review was not registered in PROSPERO. The non-registration is acknowledged straightforwardly as a limitation of the present review. All methodological decisions were nonetheless pre-specified *a priori*, and the completed PRISMA-DTA checklist is provided in Supplementary Materials as a transparency measure (Supplementary S2).

The research question was structured according to the Population—Index test—Outcome (PIO) framework for diagnostic test accuracy reviews, which is more appropriate than PICO, which is designed for intervention studies:

Are skeletal methods of age estimation based on radiographic maturation of wrist-hand (index test-I) reliable for the estimation (outcome-O) of the adult age for forensic purposes (population-P)?
**PIO COMPONENTS**
**POPULATION (P)**Adolescents and young adults in the transition to adulthood (≥16 years) undergoing forensic age assessment for legal, immigration, asylum, or criminal justice purposes.**INDEX TEST (I)**Wrist-hand skeletal maturation assessment using radiographic methods, including the Greulich-Pyle (GP) atlas method, Tanner-Whitehouse methods (TW2, TW3), automated/artificial intelligence methods (BoneXpert, deep learning, machine learning algorithms), epiphyseal fusion assessment methods, and combined or modified approaches.**OUTCOMES (O)**–primary outcomes include diagnostic accuracy metrics at specific legal age thresholds (16, 18, 21 years) considering sensitivity, specificity, positive and negative predictive values (PPV, NPV), accuracy, area under the receiver operating characteristic curve (AUC), receiver operating characteristic (ROC) curve, likelihood ratios (LR+, LR−), diagnostic odds ratio (DOR);–secondary outcomes include method accuracy measures, considering mean absolute error (MAE), mean squared error (MSE), root mean squared error (RMSE), correlation coefficients (Pearson *r*, Spearman *ρ*), concordance correlation coefficient, and limits of agreement.–tertiary outcomes include reliability measures: inter-rater reliability (intraclass correlation coefficients-ICC, and Cohen’s kappa), agreement statistics.

The reference standard for outcome evaluation was chronological age determined from verified documentation (birth certificates, official government records, or other reliable documentation).

Studies were eligible only when fully meeting the PIO-based criteria. Original research articles, including observational cohort studies (prospective or retrospective), cross-sectional studies, validation studies, method comparison studies, and diagnostic accuracy studies reporting quantitative outcomes, were considered.

### 2.2. Data Sources and Search Strategy

Six electronic databases were systematically searched from January 1980 to January 2026: Scopus (Elsevier), PubMed/MEDLINE (National Library of Medicine), Google Scholar (Google), Embase (Elsevier), Cochrane Library (Wiley), and Web of Science (Clarivate).

A comprehensive Boolean search strategy was developed in consultation with an information specialist, combining three concept groups:

“forensic age estimation” OR “age determination” OR “age verification” OR “legal age” OR “chronological age” OR “biological age” OR “age assessment” OR “adult age” OR “adult threshold” OR “adulthood” OR “18 years”
AND
“skeletal maturation” OR “skeletal development” OR “skeletal age” OR “bone age” OR “ossification” OR “epiphyseal fusion” OR “skeletal growth”
AND
“wrist-hand” OR wrist OR hand OR carpal OR metacarpal OR phalanx OR radius OR ulna OR “Greulich-Pyle” OR “Tanner-Whitehouse” OR radiography OR “X-ray” OR “artificial intelligence” OR “machine learning” OR “deep learning” OR “BoneXpert”.

Search strategies were adapted for each database’s syntax and controlled vocabulary (Medical Subject Headings (MeSH) terms for PubMed, Emtree terms for Embase). Complete search strategies for all databases are provided in Supplementary Materials—Supplementary S1. The search strategy was validated by ensuring retrieval of 10 key articles known a priori to meet inclusion criteria.

### 2.3. Study Selection

Study selection followed a two-stage screening process conducted independently by two reviewers. All identified records were imported into reference management software (EndNote, Zotero). Duplicates were identified and removed using automated and manual methods. Stage 1 consisted of title and abstract screening; Stage 2 consisted of full-text screening of included studies. Inter-rater agreement was calculated using Cohen’s kappa statistic.

### 2.4. Data Collection and Extraction

Data extraction was conducted independently by two trained reviewers using a standardized, piloted data extraction form (Supplementary Materials—Supplementary S4). The form was developed based on PRISMA-DTA recommendations [27] and piloted on five included studies before full implementation.

Data extraction followed eight predefined categories according to Table 1:

Methodological quality and risk of bias were assessed independently by two reviewers using the Quality Assessment of Diagnostic Accuracy Studies-2 (QUADAS-2) tool [24].

The primary effect measures for threshold-specific diagnostic accuracy were those specified in Table 1, column 6. The primary effect measures for overall accuracy were those specified in Table 1, column 5.

### 2.5. Statistical Analysis and Meta-Analysis

Quantitative meta-analysis was conducted for the primary and secondary outcomes.

Meta-analysis 1: diagnostic accuracy at the age 18 threshold was calculated using a DerSimonian-Laird random-effects meta-analysis [28] to estimate the method of moments for between-study variance (τ^2^). Sensitivity and specificity were pooled separately on the proportion scale with a continuity correction of 0.5 applied to extreme values (0% or 100%). Pooled estimates with 95% confidence intervals were calculated using inverse-variance weighting with random-effects adjustment. The summary receiver operating characteristic (SROC) curve was constructed using the Moses-Littenberg method. This approach was validated using the R metafor package (DL method, proportion scale) [29], confirming the reproducibility of all reported estimates. Alternative approaches (logit-scale DL, bivariate maximum likelihood) yielded higher estimates but were not used due to reduced interpretability and potential overestimation given the very high heterogeneity.

Meta-analysis 2: MAE was calculated using the DerSimonian-Laird random-effects meta-analysis model [28] to estimate the method of moments for between-study variance (τ^2^) (pooled MAE with 95% confidence interval, between-study variance (τ^2^)). Python 3.x with NumPy and SciPy libraries was used.

For both meta-analyses, heterogeneity was quantified using the I^2^ statistic [30], Cochran’s Q test, τ^2^, and H^2^ statistic. When substantial heterogeneity was detected (I^2^ > 50%), pre-specified subgroup analyses were conducted to explore potential sources of variation by estimation method (GP, TW2/TW3, AI/ML) and population type (forensic vs. clinical) using the Q-test between subgroups. Univariate and multivariate meta-regression analyses were performed to quantify the proportion of heterogeneity explained by pre-specified covariates. Residual heterogeneity unexplained by covariates was reported as the remaining proportion of I^2^ after model fitting. Where heterogeneity remained extreme and unexplained, pooled estimates were interpreted with caution, and the prediction interval (PI) for new study results with the 95% CI was reported to convey the expected range of true effects in a new, independent population/study.

Sensitivity analyses were conducted to assess the robustness of findings by excluding studies at high risk of bias, studies with small sample sizes (*n* < 100), outlier studies (standardized residuals > 2), and by comparing alternative meta-analysis models (fixed-effect vs. random-effects).

### 2.6. Reporting Bias and Certainty of Evidence Assessment

Publication bias and small-study effects were assessed using funnel plots (effect size vs. standard error), Egger’s test (statistical test for funnel plot asymmetry [31]), and Deeks’ test (funnel plot asymmetry test specific to diagnostic accuracy studies [32]). Certainty of evidence was assessed using the Grading of Recommendations Assessment, Development and Evaluation (GRADE) framework adapted for diagnostic test accuracy studies [33,34]. Risk of bias was assessed using QUADAS-2 results: inconsistency of results was assessed using heterogeneity statistics (I^2^); indirectness of study evidence was assessed by comparing study populations, index tests, and outcomes to the review question; precision was assessed using sample size and confidence interval width; and publication bias was assessed using funnel plots and statistical tests.

## 3. Results

### 3.1. Study Selection and Inclusion

**Figure 1:**
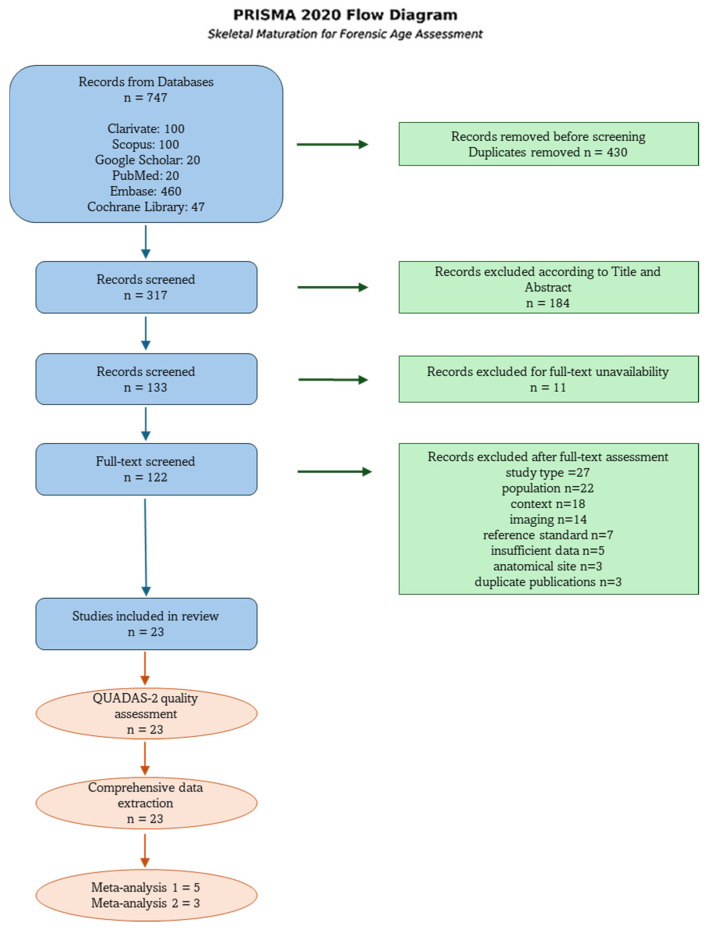
PRISMA 2020 flow diagram. Color legend: Identification in blue; Exclusion in green; Data analysis in orange.

The systematic search identified 747 records across six databases (Scopus, PubMed, Google Scholar, Embase, Cochrane Library, and Clarivate). Following the removal of 430 duplicates, 317 unique records underwent title and abstract screening. Title and abstract screening excluded 184 records that clearly did not meet eligibility criteria, yielding 133 records for full-text retrieval. Of these, 11 full-text articles could not be retrieved despite attempts to contact authors and requests through interlibrary loan services. Full-text assessment of the 122 retrieved articles resulted in the exclusion of 99 articles (Figure 1). Ultimately, 23 studies met all eligibility criteria and were included in the systematic review [35,36,37,38,39,40,41,42,43,44,45,46,47,48,49,50,51,52,53,54,55,56,57]. All 23 studies underwent QUADAS-2 quality assessment and comprehensive data extraction. Five studies provided sufficient data for meta-analysis 1 (diagnostic accuracy at the 18-year age threshold) [36,37,42,43,57], and three studies contributed data to meta-analysis 2 (MAE) [35,36,47,49,52].

The study selection process is presented in the PRISMA 2020 flow diagram (Figure 1). Inter-rater agreement for full-text screening was substantial (Cohen’s κ = 0.82, 95% CI: 0.74–0.90).

### 3.2. Characteristics of the Included Studies

Descriptive synthesis from the 23 selected studies is detailed in Table 2.

#### 3.2.1. Demographic Data

Publication years ranged from 2003 to 2026, with a notable increase after 2010 (*n* = 18, 78.2%) [35,36,37,38,39,40,41,42,43,44,45,46,47,48,49,50,51,52]. The 23 studies included a total of 11,425 participants, with sample sizes ranging from 114 participants in Garamendi et al. 2003 [57] to 2038 participants in Thodberg et al. 2017 [43], with a median sample size of 453 participants (interquartile range-IQR: 200–688). Only five studies presented samples of fewer than 200 participants [39,44,50,56,57], but none of these reported error estimates of MAE or diagnostic accuracy except for Garamendi et al. [57], which specifically focused on subjects aged 16–19 years. The majority of studies were conducted in clinical contexts (clinical/hospital or community-based populations, *n* = 13, 56.5%) [37,39,40,41,42,43,45,49,50,51,52,53,56]. Samples encompassed 15 countries spanning four continents and representing diverse geographic and ethnic populations, with European studies predominating (56.5%) [35,36,40,42,43,46,48,49,51,52,53,54,55].

#### 3.2.2. Age Cohorts

Age ranges varied considerably across studies, reflecting different research objectives and target populations. Studies with clinically sampled populations represented the broadest age ranges [41,45] and exhibited different distributions across age cohorts of interest, producing fewer subjects older than 16 years [35,36,38,39,40,41,44,45,46,47,48,49,50,51,52,53,54]. All included studies employed documented chronological age as the reference standard.

#### 3.2.3. Sex-Specific Results

Sex distribution was generally balanced across studies, with male participants comprising 47.7–53.3% of samples, except for Garamendi et al. [57], which presented an exclusively male sample (100%). Seven studies reported sex-specific analyses [36,38,41,42,43,47,52]. Pereira et al. [36] found slightly better performance of the LR 16 bones Greulich-Pyle(GP)-based method in males (MAE 1.47 years) compared to females (MAE 1.53 years), and conversely, better GP performance in females (MAE 1.39 years) compared to males (MAE 1.88 years). However, the authors did not report the respective standard deviations. Maggio et al. 2018 [41] obtained a narrower MAE range in females (±0.45 years) than in males (±0.90 years), but a wider accuracy range in females (±0.28–5.58 years) than in males (±0.00–3.53 years). Mohammed et al. 2015 [47] demonstrated similar MAE values between males and females. Tisè et al. 2011 [52] revealed superior performance for males compared to females.

#### 3.2.4. Age Estimation Methods

The GP atlas method was the most frequently evaluated approach, appearing in 17 studies (73.9%) [35,36,39,40,41,42,43,47,48,49,51,52,53,54,56,57]. Tanner-Whitehouse methods (TW2 and TW3) were assessed in five studies (21.7%) [35,37,40,45,48]. Artificial intelligence and machine learning approaches were evaluated in three recent studies (13.0%), including BoneXpert automated bone age assessment [43], convolutional neural network (CNN)-based automated TW3 assessment [37], and machine learning-based skeletal maturation prediction [35].

#### 3.2.5. Repeatability and Accuracy

Twelve studies (52.2%) provided complete data for inter-rater agreement [36,37,38,39,41,42,45,47,48,49,52,57]. High inter-rater reliability (ICC or κ > 0.90) was reported in eight studies: Pereira et al. 2026 [36], Wang et al. 2025 [37], Alcina et al. [42], Mohammed et al. 2015 [47], Pinchi et al. 2014 [48], De Donno et al. 2013 [49], Tisè et al. 2011 [52], and Garamendi et al. 2003 [57]. Conversely, Malkoçoğlu et al. 2025 reported an ICC of 0.878 for males [38], Behera et al. 2023 (κ = 0.72) [39], Maggio et al. 2018 (κ = 0.887) [41], and Maggio et al. 2016 (κ = 0.869–1.00) [45]. Notably, Malkoçoğlu et al. 2025 found very low inter-rater agreement for females (ICC = 0.404) [38].

Results presentation in terms of accuracy was highly variable, with most studies presenting solely residual analyses without standard measurements and showing different results according to cohort population type. Several authors reported a significant trend toward overestimation around the 17-year adult threshold when applying GP and underestimation at the 18-year age threshold [42,48,52], whereas others demonstrated an overestimation trend solely in females [47] or in all participants aged 17–19 years estimated as 19 years according to the GP atlas [56]. Overestimation in females applying TW2 was observed [40], whereas an underestimation trend was noted when applying TW3 [48]. Thodberg et al. 2017 [43], validating their AI-based BoneXpert GP-based system, set an a priori threshold of 19.0 years to reduce the false-positive rate to 2%, as a threshold of 18.5 years resulted in a 7% false-positive rate.

Five studies [35,36,47,49,52] reported the accuracy of estimation methods, revealing high variability, with MAE ranging from a maximum of 1.88 years in males using GP [36] to a minimum of 0.27 [49], and standard deviations (SDs) even from the same study varying from ±0.27 to ±2.12 years [49]. Overall, heterogeneity in MAE result presentation was evident, considering that only three also reported the standard deviation of the measured error [35,49,52], whilst Maggio et al. 2018 [41] only reported the SD of the MSE (Mean Squared Error). Diagnostic accuracy at the 18-year age threshold in terms of sensitivity, specificity, PPV, NPV, accuracy, and AUC was reported in five studies [36,37,42,43,57], demonstrating high variability across expert raters. Specificity using GP at both the 17-year and 18-year age thresholds varied from 64% to 100%, whereas sensitivity ranged from 45% to 97% [36,42,57]. Sensitivity and specificity measured when estimating age with TW3 varied between 36–46% and 83–97%, respectively [37]. The AI method based on automated TW3 CNN demonstrated significantly superior performance compared to manual assessment, particularly for sensitivity [37].

### 3.3. Risk of Bias Assessment

**Figure 2:**
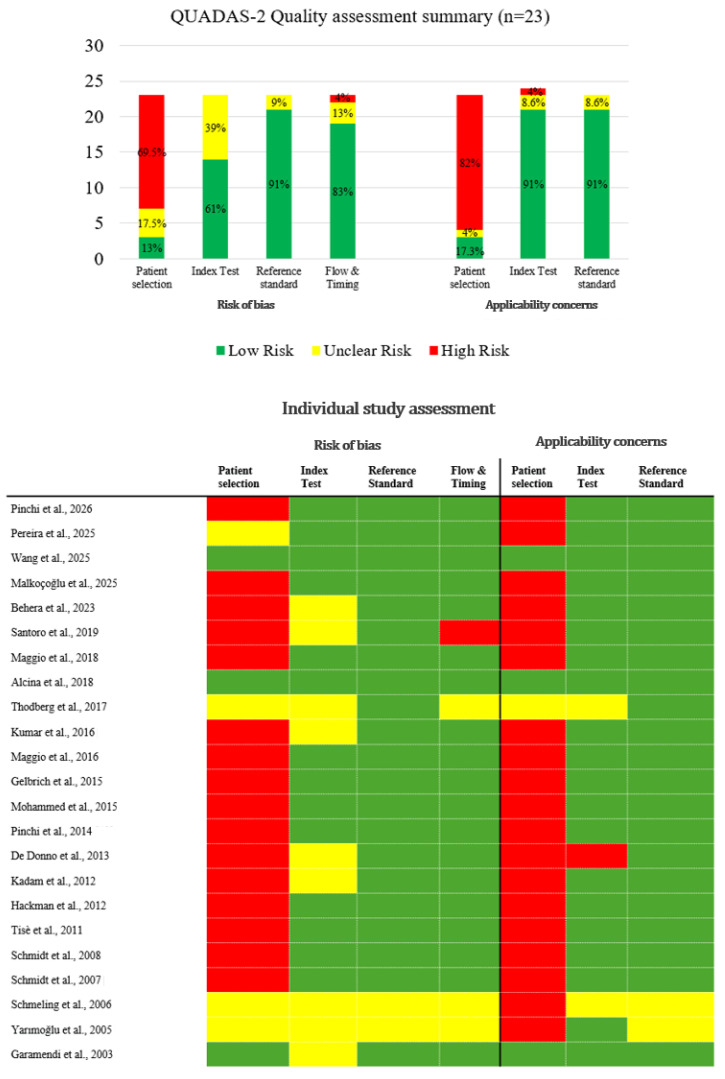
QUADAS-2 quality assessment of included studies (*n* = 23). The top panel shows summary risk of bias and applicability concerns across all domains, with stacked bars indicating the *n*. of studies rated as low risk/concern (green), unclear (yellow), or high risk/concern (red). The bottom panel displays individual study assessments with color-coded squares for each domain. Studies are ordered by descending publication year (2026–2003). Manuscript references are [35,36,37,38,39,40,41,42,43,44,45,46,47,48,49,50,51,52,53,54,55,56,57], starting from the top.

Quality assessment using the QUADAS-2 tool revealed variable methodological quality across the 23 included studies (Figure 2). Most studies (82.6%) exhibited high or unclear patient selection bias, with only three studies (13.0%) rated as low risk [37,42,57], characterized by careful description of sample selection procedures and age cohorts adequate for studying the 18-year age threshold. The use of non-forensic populations and clinical sampling methods that resulted in unevenly distributed age cohorts represented the main issues identified in 16 studies (69.5%) considered to be at high risk of bias [35,38,39,40,41,44,45,46,47,48,49,50,51,52,53,54]. In a further four studies (17.4%), the risk of bias was unclear [36,43,55,56]. Therefore, all these studies raise relevant and substantial concerns regarding applicability to forensic practice due to high or unclear risk of bias.

In the index test domain, nine studies (39.1%) were rated as unclear risk [39,40,43,44,49,50,55,56,57] due to unclear blinding to chronological age during skeletal maturity assessment and inadequate assessment of measurement reliability. Conversely, the reference standard domain demonstrated excellent quality, as did the flow and timing domain.

### 3.4. Diagnostic Accuracy Meta-Analysis at Age 18 Threshold

Meta-analysis 1 for diagnostic accuracy included five studies [36,37,42,43,57] encompassing 9888 assessments from 4587 samples. The studies provided sufficient data for evaluating overall diagnostic accuracy at the 18-year age threshold according to sensitivity, specificity, accuracy, positive predictive value (PPV), negative predictive value (NPV), and area under the curve (AUC), based on diverse age estimation methods including the GP atlas [36,42,57], linear regression using GP 16-bone assessment [36], manual and automated TW3 assessment by multiple experts and CNN models [37], and automated GP-based AI (BoneXpert) [43] (Table 2).

**Figure 3:**
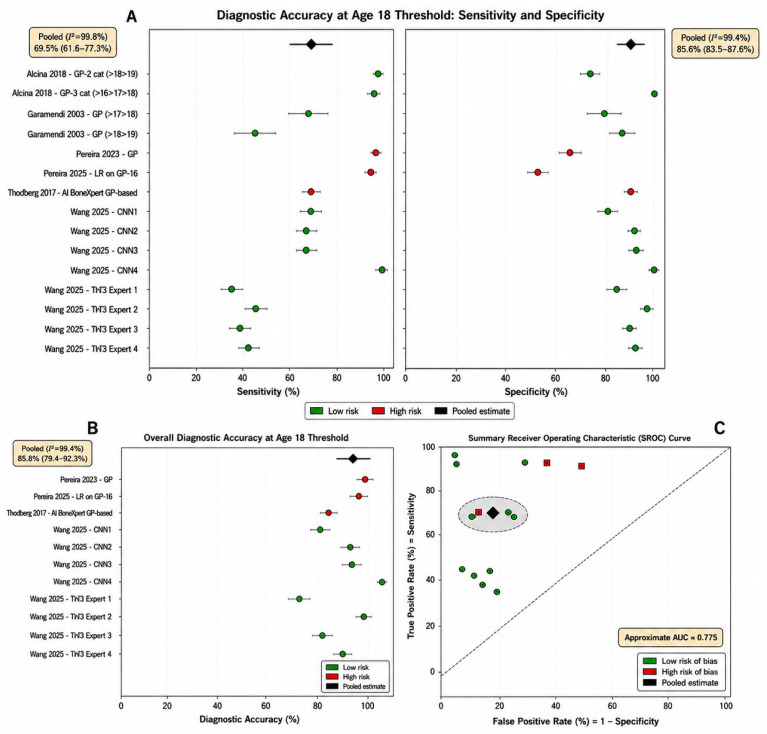
Panel (**A**) shows a coupled pooled sensitivity-specificity forest plot: side-by-side comparison demonstrating the trade-off between sensitivity and specificity across studies. The pooled estimate is shown as a black diamond with 95% CI. High-risk studies (red circles) tend to cluster at higher sensitivity values, whereas low-risk studies (green circles) show more conservative estimates. Panel (**B**) highlights random-effects meta-analysis of overall accuracy (% correctly classified). Individual study estimates are shown as circles with 95% confidence intervals (error bars); circle colors indicate risk of bias (green = low, red = high). The pooled estimate is shown as a black diamond with 95% CI. Panel (**C**) displays a summary receiver operating characteristic (SROC) curve across all studies: the pooled estimate (black diamond) is at a sensitivity of 0.69 and a specificity of 0.85 (false-positive rate [FPR] = 0.13), with the gray oval region showing the uncertainty. Both high-risk studies (red circles) and low-risk studies (green circles) are dispersed. The dashed diagonal line represents chance performance (AUC = 0.5). Note: The CNN4 accuracy was adjusted from 100% to 99.5% for continuity correction.

The DerSimonian-Laird random-effects model yielded a pooled sensitivity of 69.5% (95% CI: 61.6–77.3%), specificity of 85.6% (95% CI: 83.5–87.6%), pooled AUC of 0.775, and accuracy of 85.9% (95% CI: 79.4–92.3%) (Figure 3).

Extremely high heterogeneity (I^2^ = 99.8% for sensitivity, I^2^ = 99.4% for specificity, and I^2^ = 99.4% for accuracy) demonstrates substantial variability in diagnostic classification across studies. The respective PIs for future studies’ diagnostic reliability, calculated with 95% CI, are 35.3–100% (sensitivity), 77.2–93.9% (specificity), and 60.3–100% (accuracy).

The included studies permitted subgroup analysis to explore sources of variation.

**Figure 4:**
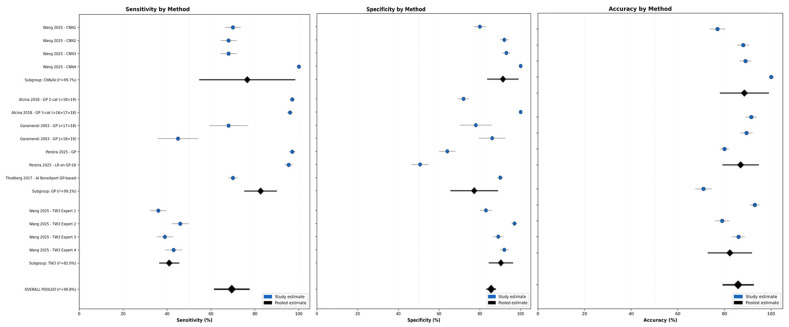
Forest plot of sensitivity (**left**), specificity (**middle**), and accuracy (**right**) of individual studies pooled by assessment method. The black diamond represents the pooled measure.

**Figure 5:**
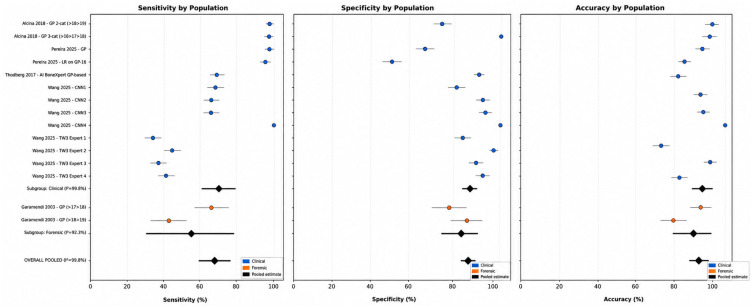
Forest plot of sensitivity (**left**), specificity (**middle**), and accuracy (**right**) of individual studies pooled by population type.

Subgroup diagnostic accuracy meta-analysis at the 18-year age threshold according to the different methods used to provide estimates and population type (clinical vs. forensic samples) is shown in Table 3 and Figure 4, and in Table 4 and Figure 5, respectively.

Among automated methods, CNN-based machine learning approaches demonstrated promising results, outperforming traditional manual methods for specificity and accuracy (91.3% and 88.6%, respectively). Among traditional methods [36,42,57], TW3 demonstrated unacceptably low sensitivity, missing more than half of adults at the 18-year threshold (41.0%). GP was the most commonly analyzed method, demonstrating high sensitivity but lower specificity, with more minors misclassified as adults.

Clinical populations demonstrated improved pooled sensitivity of +14.8 percentage points compared to forensic populations, though this comparison is limited by the small forensic sample (one study, *n* = 114). Conversely, the small difference in percentage points (+3.6) between pooled specificities is negligible, representing similarity across population types. High heterogeneity characterized studies based on clinical samples (I^2^ > 99% for both outcomes), attributable to diverse methods (manual GP, automated BoneXpert, AI-based CNN), geographic settings (China, Portugal, Spain, multi-country), and age thresholds enacted by law.

**Figure 6:**
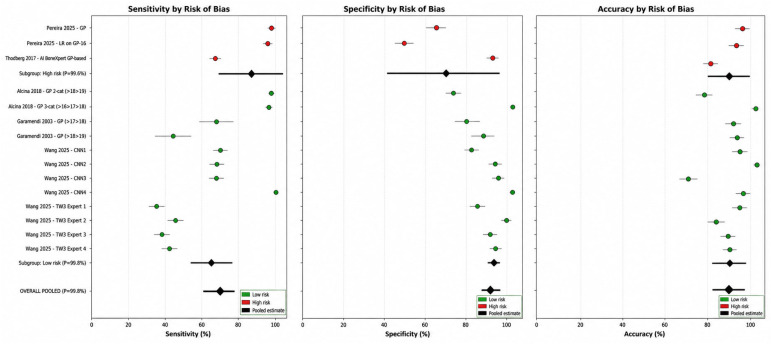
Forest plot of sensitivity test results according to QUADAS-2 assessment, excluding high-risk studies from pooled diagnostic accuracy measures. Comparison of sensitivity results (**left**), specificity (**middle**), and accuracy results (**right**) across studies.

According to the QUADAS-2 quality assessment, only three of the five studies applicable for meta-analysis reported a low risk of bias [37,42,57]. Restricted sensitivity, specificity, and accuracy analyses yielded similar results overlapping with overall meta-analysis results, with differences of +4.6, − 4.3, and +0.4 percentage points, respectively. The substantial overlap in confidence intervals indicates that diagnostic accuracy results are reasonably robust to the exclusion of high-risk studies (Table 5 and Figure 6).

Comparison of pooled measures between low-risk [37,42,57] and high-risk studies [36,43] revealed that high-risk estimation demonstrated inflated sensitivity (87.5% vs. 64.8%) but deflated specificity (68.3% vs. 89.9%), with overall accuracy similar between the two. This result suggests that sensitivity and specificity errors in pooled measures may cancel each other out and balance the obtained overall accuracy value.

Heterogeneity remained very high even within low-risk studies (I^2^ = 99.8% for sensitivity, I^2^ = 99.0% for specificity, and I^2^ = 99.3% for accuracy), indicating that study quality alone does not fully explain the observed variability.

Publication bias assessment according to Deeks’ funnel plot asymmetry test was not assessable due to insufficient data with complete 2 × 2 contingency tables (true positives [TP], false positives [FP], true negatives [TN], false negatives [FN]). Nevertheless, coupled forest plots suggest potential asymmetry related to small-study effects, with smaller studies tending toward higher sensitivity estimates.

### 3.5. Mean Absolute Error (MAE) Meta-Analysis

Meta-analysis 2 for overall accuracy included 11 estimated values from three studies [35,49,52]. Two studies [36,47] were excluded due to missing standard deviation data required for inverse-variance weighting. Two additional studies by Maggio et al. 2018 [41] and Thodberg et al. 2017 [43] provided only prediction intervals but not MAE values, thus being excluded from the outset. The included studies evaluated diverse age estimation methods, including the GP atlas [35,49,52], manual TW3 assessment [35], and automated GP/TW3 machine learning [35] (Table 2).

**Figure 7:**
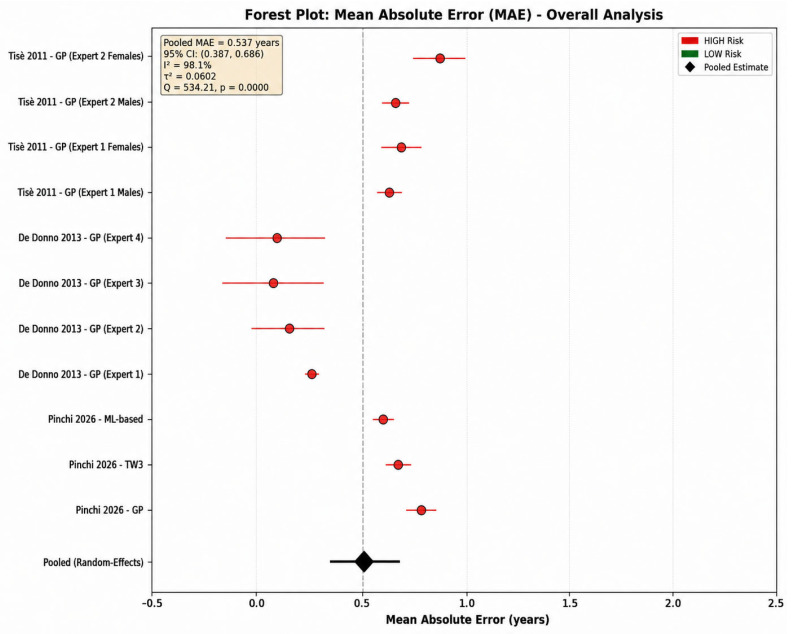
Forest plot showing the mean absolute error (MAE) of skeletal maturation-based age estimation across three included studies with 11 estimated values. Mean errors and prediction ranges are represented by circles and bars. The black diamond represents the pooled measure.

Using the DerSimonian-Laird random-effects model yielded an overall pooled MAE of 0.537 years (95% CI: 0.387–0.686, SE 0.08 years), equivalent to 6.4 months average error (Figure 7).

Heterogeneity in MAE estimates was very high (I^2^ = 98.1%, Q = 534.2, *p* < 0.001), indicating relevant inconsistency across studies. The calculated PI with 95% CI for future studies’ errors is 0.33–0.79 years.

**Figure 8:**
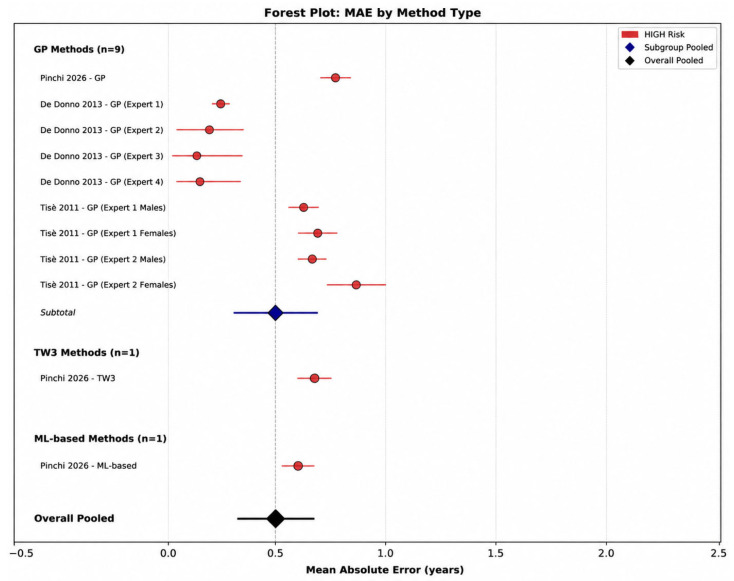
Forest plot of pooled MAE by assessment method.

Subgroup analyses of MAE according to assessment methods are shown in Table 6 and Figure 8.

GP methods represent the most commonly used tool, yielding a pooled MAE of 0.503 years (6.0 months) [35,36,47,49,52] and demonstrating the best performance. However, the comparison is limited by the small number of TW3 and ML-based estimates.

Subgroup MAE meta-analysis according to population type (clinical vs. forensic samples) was not feasible because all three included studies sampled participants and radiographs in clinical contexts and limited the research to Italian populations [35,49,52].

According to QUADAS-2 quality assessment, all 11 estimates included in the meta-analysis (with SD data) originated from high-risk studies [35,49,52]. Thus, sensitivity analysis by risk of bias was not feasible.

**Figure 9:**
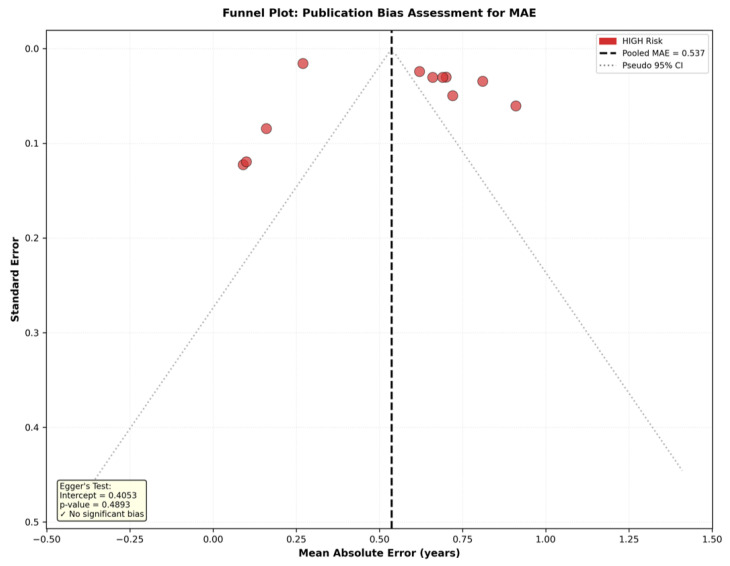
Funnel plot inspection of MAE meta-analysis publication bias shows the distribution of the considered high-risk studies (red circles).

Publication bias assessment according to Egger’s regression test for funnel plot asymmetry indicated no significant publication bias (intercept = 0.405, *p* = 0.489), and visual inspection of the funnel plot revealed a symmetrical distribution of estimates around the pooled MAE (Figure 9). This suggests that observed results are unlikely to be substantially affected by selective reporting.

A meta-regression analysis of MAE heterogeneity was conducted to better understand the nature of this high value (Table 7).

**Figure 10:**
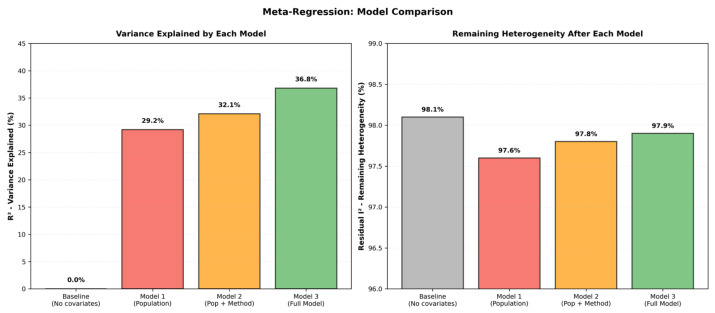
Comparison among tested multivariate meta-regression models.

Population type was the strongest predictor of MAE heterogeneity (R^2^ = 29.1%), with forensic contexts yielding +0.47 years higher MAE than clinical contexts. The full model explains 36.8% of heterogeneity (population + method + sample size, Figure 10), but 63.2% remains unexplained.

### 3.6. Certainty of Evidence Assessment (GRADE)

**Figure 11:**
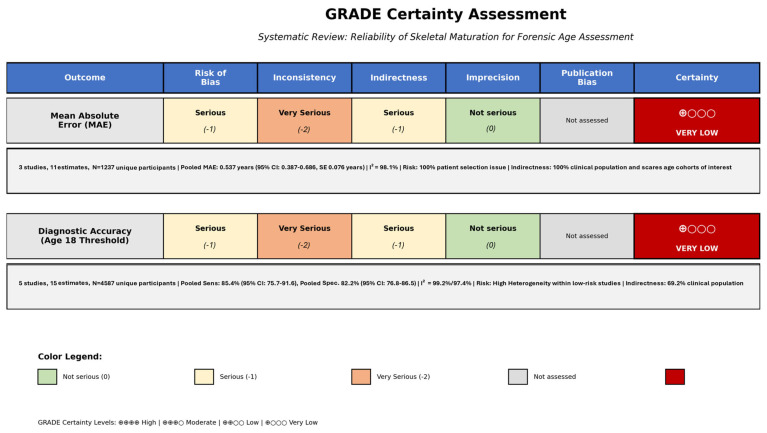
GRADE Certainty Assessment for skeletal maturation diagnostic accuracy and measurement precision. Both outcomes received VERY LOW certainty (⊕○○○) due to serious risk of bias (-1), very serious inconsistency (-2), and serious indirectness (-1). Color coding: green = not serious (0), yellow = serious (-1), orange = very serious (-2), grey = not assessed.

The Grading of Recommendations Assessment, Development and Evaluation (GRADE) approach was applied to assess the certainty of evidence for the two primary outcomes: diagnostic accuracy (sensitivity and specificity) at the 18-year age threshold and mean absolute error (MAE) in age estimation (Figure 11).

According to the VERY LOW certainty definition, we have very little confidence in the effect of any estimate (both accuracy at the age threshold and MAE), which is very uncertain, and true precision likely differs substantially and varies [58].

A summary of the main findings of meta-analyses 1 and 2 is reported in Table 8.

## 4. Discussion

This systematic review and meta-analysis synthesized evidence from 23 studies (11,425 participants, 15 countries) evaluating wrist-hand radiographic skeletal maturation methods for forensic age assessment, particularly at the 18-year age threshold. The review yielded several key findings with important implications for forensic practice in young adult age estimation. Two complementary meta-analyses examined diagnostic accuracy (13 estimated values from five studies, 9888 assessments) and mean absolute error (11 estimated values from three studies, 4495 assessments). The pooled diagnostic accuracy at the 18-year threshold demonstrated very low sensitivity of 69.5% (95% CI: 61.7–77.3%) and higher specificity of 85.6% (95% CI: 83.5–87.6%), with an overall accuracy of 85.9% (95% CI: 79.4–92.3%). While these results could suggest reasonably good accuracy, the 95% prediction intervals (35.3–100% for sensitivity, 77.2–93.9% for specificity, and 60.3–100% for accuracy) reveal that performance in a new study population may vary substantially from these pooled values. For example, a new study could plausibly find sensitivity ranging from very low (~35%) to perfect performance (100%), thus paradoxically ranging from useless to acceptable evidence for forensic age estimation with the same probability, the same results forensics have to present to Courts. The observed I^2^ values exceeding 98% for all diagnostic accuracy outcomes deserve explicit methodological comment. High I^2^ does not invalidate pooled estimates; rather, it indicates that the true effect varies substantially across studies inter-variability and that the pooled estimate represents a weighted average of genuinely different effects. In high-heterogeneity settings, the 95% confidence interval quantifies uncertainty around this average, while the 95% prediction interval quantifies the expected range for the true effect in a new study. This wide variability is a direct consequence of extreme between-study heterogeneity (τ = 0.15 for sensitivity) and should not be conflated with imprecision in the pooled estimate itself, but rather meaningful summaries of the central tendency of a heterogeneous body of evidence. However, the corresponding prediction intervals (sensitivity 35.3–100%, specificity 77.2–93.9% with 95% CI) confirm that no single study result should be expected to replicate the pooled estimate exactly, and that the practical utility of wrist-hand skeletal maturation methods varies substantially with population, methodology, and context. This interpretation is consistent with the GRADE VERY LOW certainty rating, which reflects not only heterogeneity but also pervasive risk of bias and serious indirectness. Anyway, the 70% sensitivity means that approximately one in three adults (30%) are incorrectly classified as minors, potentially denying them adult legal rights or protections. The 85% specificity means that nearly one in seven minors (15%) are misclassified as adults, exposing them to inappropriate legal treatment or prosecution as adults. In hypothetical forensic contexts involving 1000 individuals (500 minors, 500 adults), these accuracy estimates would result in approximately 150 adults incorrectly classified as minors and 75 minors incorrectly classified as adults. The moderate AUC of 0.775 falls short of the ≥0.90 threshold typically considered excellent for diagnostic tests, indicating substantial overlap in skeletal maturity between individuals just below and above 18 years. In the context of a binary legal decision (minor vs. adult), the AUC represents the probability that the test correctly rank-orders a randomly selected true minor against a randomly selected true adult. An AUC of 0.775 therefore means that in approximately 1 in every 4–5 paired comparisons between a true minor and a true adult near the 18-year boundary, the test assigns a higher skeletal age to the minor than to the adult, the wrong rank ordering for a binary legal classification. To better explain, a coin flip yields an AUC of 0.50, so the observed AUC of 0.775 should be interpreted as evidence that skeletal maturation of the wrist-hand provides very low discriminatory capacity at the legally critical 18-year boundary, carrying substantial individual-level risk not acceptable as evidence in Courts. Such misclassification rates have serious legal and ethical implications since the legal threshold of 18 years distinguishes juvenile from adult jurisdiction and directly affects criminal, civil, and immigration procedures. Misclassification of minors may result in violations of international human rights protections, including exposure to adult detention and punitive sentencing. In asylum contexts, incorrect age assessment can deny unaccompanied minors access to guardianship, specialized care, and procedural safeguards. On the other hand, attainment of legal majority also determines civil capacities, including autonomous medical consent, marriage, voting rights, and contractual competence (for instance obtaining a legal job). In addition, forensic practitioners involved in age estimation have the deontological and ethical responsibility of guaranteeing the legal principles of autonomy, beneficence, non-maleficence, and justice, prioritizing the best interests of the presumed minor and selecting reliable and evidence-based approaches to protect fundamental rights of both minors and adults [19,23].

In this context, it should be noted that authors of some included studies [40,42,43,47,48,52,56] reported significant trends in under- and overestimation around the adult threshold when applying GP, TW2, TW3, and BoneXpert GP-based methods to their samples. Specifically, Santoro et al. 2019 [40] reported TW2 limitations for female bone maturity scores, yielding significant rates of age overestimation up to 2 years in both Italian and African samples, whereas underestimation occurred in the lower age range for males. Alcina et al. 2018 [42] reported a mean overestimation of +0.32 years in subjects aged 17 years and a mean underestimation of −0.23 years in subjects aged 18 years using the GP method. Thodberg et al. 2017 [43], validating their AI-based BoneXpert GP system, set an a priori threshold of 19.0 years to reduce the false-positive rate to 2%, as a threshold of 18.5 years resulted in a 7% false-positive rate. Mohammed et al. 2015 [47] found that skeletal age evaluated through GP in males was underestimated by 0.23 ± 1.53 years and overestimated by 0.02 ± 2.00 years in females, with most female children demonstrating complete maturity of hand bones at 17 years, in contrast to male children (bone maturity at 19 years). Pinchi et al. 2014 [48] reported underestimation for males and females aged >16 years estimated with TW3 and aged >18 years with GP and TW3, whereas an overestimation trend was observed in male and female subjects aged 16–17 years estimated with GP and TW2. According to Yarımoğlu et al. 2005 [56], the Greulich-Pyle standards determined a bone age estimation on average 0.49 years ahead of chronological age in females and 0.61 years ahead of chronological age in males. They found that all 51 cases with chronological ages of 17, 18, and 19 years had a bone age of 19 years according to their GP wrist radiographs. Furthermore, Tisè et al. 2011 [52] overestimated 22.9% of 16-year-old and 73.2% of 17-year-old males, as well as 23.5% of 16-year-old and 75.0% of 17-year-old females, when applying the GP method. They reported frequencies of under- and overestimated males in reaching adult age at the actual age of 17 years of 11 minors and 30 adults. Similarly, the frequencies of under- and overestimated females in reaching adult age at the actual age of 17 years were three minors and nine adults.

The overall mean absolute error across methods was 0.537 years (95% CI: 0.387–0.686 years), though this estimate exhibited very high heterogeneity (I^2^ = 98.1%, *p* < 0.001), indicating that 98.1% of the observed variance in error estimates reflects true differences between studies rather than sampling error alone. This extreme heterogeneity suggests that pooled estimates should be interpreted with caution, as individual study results vary substantially. The 95% prediction interval for MAE (0.33–0.79 years) quantifies this uncertainty, suggesting that in a new study, the expected MAE could plausibly range from approximately 4 to 10 months, even if the pooled estimate is 6.4 months. Meta-regression analysis identified population type applicability as the strongest predictor of MAE variability, explaining 29.1% of heterogeneity. Forensic contexts demonstrated significantly higher error rates (+0.47 years, *p* < 0.001) compared to clinical contexts, thus reflecting the fact that all three included studies were actually conducted on Italian clinical populations. A full model incorporating population contexts, assessment method, and sample size explained 36.8% of the observed heterogeneity, leaving 63.2% attributable to unmeasured factors such as ethnicity, health status, rater experience, radiograph quality, and, most importantly, inadequate and heterogeneous age cohort distributions. This large unexplained variance underscores fundamental gaps in understanding what drives differences in accuracy across settings. It must also be emphasized that the evaluated MAEs are not specifically calculated for the 18-year threshold estimation, as no studies provided the error measures precisely requested for this review. Conversely, the three studies included in the MAE meta-analysis [35,49,52] were classified as high risk of bias precisely due to poor representation of enrolled subjects >18 years old, thus precluding generalization of evidence. The pooled estimate therefore characterizes a narrow, geographically and methodologically homogeneous subset of the literature. Forensics should be explicitly warned that the 0.537-year pooled MAE could demonstrate reasonable precision for clinical growth monitoring, but the performance of these methods can’t be absolutely generalized for forensic non-Italian populations with an age distribution relevant to the 18-year threshold determination. The pooled MAE raises serious concerns for forensic contexts where legal thresholds are absolute and binary, especially when the assessment is to be evidence-based for judicial decisions. Furthermore, the 95% confidence interval (0.387–0.686 years) spans nearly 0.3 years, meaning individual predictions could easily misclassify an individual by 4–7 months in either direction. When combined with the very high heterogeneity (I^2^ = 98.1%) and calculated PI for future studies (0.33–0.79 years), this uncertainty becomes even more problematic, suggesting that the true error in a new forensic population could be substantially higher than 0.537 years. The substantial performance gap between forensic and clinical populations (+0.47 years MAE, *p* < 0.001) is particularly concerning. This finding suggests that methods validated primarily in clinical settings (73.9% of studies) may systematically underperform when applied to forensic populations with truly blinded chronological age and specific age thresholds of interest.

QUADAS-2 assessment revealed pervasive methodological limitations across the evidence base. Patient selection bias was nearly universal. 82.6% of studies (19/23) exhibited high risk of bias due to non-consecutive or non-random sampling, inappropriate exclusions or inclusions, inadequate age cohort distributions, or case-control designs that artificially inflate accuracy. Additionally, 52.2% of studies (12/23) exhibited high risk in index test conduct (lack of blinding, threshold manipulation), and 43.5% (10/23) in reference standard application (inappropriate reference standards, lack of blinding). Critically, only two studies achieved low risk of bias across all four QUADAS-2 domains, indicating that the entire evidence base is compromised by methodological weaknesses. The “spectrum bias” is the most important threat to the validity of the pooled diagnostic accuracy estimates, as selective sampling may overestimate performance compared to real-world forensic screening scenarios. In the present review, 69.5% of included studies enrolled participants starting from childhood (ages 4–16 years), creating study populations in which the vast majority of individuals are clearly, unambiguously not yet 18 years old. When the pool’s sensitivity and specificity across such studies are considered, the specificity estimate is inflated by the inclusion of large numbers of young children who will be correctly classified as minors with near-certainty, but not because the test is accurate near the 18-year boundary, rather because the test is trivially accurate for a 14-year-old. This spectrum effect directly explains the apparent paradox between the acceptable pooled specificity (85.6%) and the VERY LOW certainty of evidence: the specificity looks reasonable at the aggregate level precisely because many included studies were not measuring the clinically relevant question. The forensic question is whether wrist-hand maturation can discriminate between a 17-year-old and a 19-year-old, not between a 7-year-old and a 25-year-old. Only studies that restrict their samples to the clinically relevant age window (approximately 15–21 years) can provide unbiased estimates of diagnostic accuracy at the 18-year threshold. Of the five studies contributing to Meta-analysis 1, the forensic relevance of the age distribution varies substantially, and this variation is a primary driver of the extreme heterogeneity (I^2^ > 98%) observed. Forensicists should be explicitly informed that the pooled specificity of 85.6% almost certainly overestimates the true specificity in a forensically relevant population of individuals aged 15–21 years.

The cumulative impact of these quality issues resulted in a GRADE certainty of evidence rated as VERY LOW (⊕○○○). This rating reflects serious risk of bias (downgraded two levels due to 82.6% high risk in patient selection and absence of low-risk studies), serious inconsistency (downgraded one level for I^2^ = 98.1% with unexplained heterogeneity), and serious indirectness (downgraded one level for nearly 100% clinical populations applied to forensic questions). Very low certainty means there is little confidence that the true effect lies close to the pooled estimates, and the true accuracy could be substantially different from what this review reports.

Sensitivity analysis excluding high-risk studies demonstrated minimal change in pooled diagnostic accuracy estimates (sensitivity 64.84% vs. 69.46%, specificity 89.90% vs. 85.56%), suggesting robustness of the diagnostic accuracy findings despite quality concerns. However, sensitivity analysis for MAE was impossible because all three studies contributing MAE data were rated as high risk of bias in at least one domain. This complete absence of low-risk MAE evidence further undermines confidence in the 0.537-year pooled estimate and highlights a critical evidence gap.

This meta-analysis has several limitations, including the unfeasibility of individual-level data analysis due to aggregate reporting, limited assessment of publication bias and sensitivity tests, and very low certainty of evidence requiring cautious interpretation. But the main substantial limitation is due to the structural mismatch between the review’s stated objective (evaluating forensic utility of skeletal age estimation methods) and its evidence base, particularly conducted on a clinical population (56.5% of included studies), not corresponding to the forensic settings, where age is blinded, populations are selected, and the stakes of misclassification are qualitatively different. This is a fundamental limitation that cannot be resolved through statistical adjustment but through further population-specific validations for forensic age cohorts and samples of interest. The main structural limitation is the inapplicability of the Deeks’ test. It requires individual study-level data (effective sample size and diagnostic odds ratio) that the majority of primary studies did not report in a compatible format. Consequently, Deeks’ test could not be performed regardless of the number of included studies, but this is more a structural reporting deficiency in the primary literature and is acknowledged as a constraint on the completeness of the publication bias assessment for diagnostic accuracy. In fact, coupled forest plots suggest potential asymmetry related to small-study effects, assuming smaller new studies tend toward higher sensitivity estimates.

Nevertheless, this review represents the only available comprehensive evidence synthesis regarding the diagnostic accuracy and reliability of skeletal methods based on radiographic assessment of wrist-hand bone maturation for estimating or classifying the age of individuals at the adult threshold (≥18 years old). Bhardwaj et al. [19], in an overview published in 2024, stated that despite wrist and hand radiographs being the most commonly performed radiographic investigation, they alone would not be adequate for all relevant age categories, including maturity age cohorts. In such cases, other skeletal sites must be considered (e.g., elbow or shoulder). Kumar et al. [44] observed that complete fusion of most wrist-hand bones used for age estimation occurs within an age range of 16–19 years, with a slight delay in males compared to females. Similar results were reported by Kadam et al. [50], who documented cases of females demonstrating complete fusion at 15 years of age.

## 5. Conclusions

Wrist-hand radiographic methods demonstrate low diagnostic accuracy (sensitivity 70%, specificity 85%) and moderate mean absolute error (0.537 years) for skeletal age assessment at the 18-year threshold, but very high heterogeneity (I^2^ > 98%), pervasive risk of bias (82.6% high risk in patient selection, 0% low risk overall), and VERY LOW certainty of evidence severely limit confidence in these estimates. The findings indicate that approximately 15–30% of individuals will be misclassified, with potentially serious legal consequences.

Nevertheless, the 95% prediction intervals (sensitivity 35.3–100%, specificity 77.2–93.9%, MAE 0.33–0.79 years) indicate that performance in a new study population may differ substantially from these pooled values, and in 63.2% of cases the observed heterogeneity remains unexplained after accounting for population, method used to estimate bone age, and sample size, suggesting that undetected or undetectable factors largely affect accuracy.

The VERY LOW certainty of evidence reflects these findings, meaning that the true accuracy will be substantially different from pooled estimates. This must induce experts to exercise extreme caution in forensic applications where misclassifications have serious legal consequences.

The present review provides the best available evidence synthesis for forensic purposes, but that evidence base is predominantly clinical. Forensic practitioners should not assume that clinical performance estimates are directly transferable to forensic settings, where age is blinded, populations are selected, and the stakes of misclassification are qualitatively different.

Future studies should prioritize population-specific validation in forensic age cohorts of interest, using consecutive sampling and appropriate reference standards to address the patient selection bias issue, reporting complete error measures.

Machine learning approaches with external validation in independent forensic populations and transparent reporting of training/validation datasets to avoid the patient selection biases plaguing current evidence should be improved.

Given the 15–30% misclassification rates and VERY LOW certainty, forensic experts should recognize the substantial limitations of current evidence on wrist-hand radiographs when making decisions affecting individual liberty, asylum status, or criminal accountability. Until higher-certainty studies emerge, relying exclusively on skeletal indicators for the assessment of the 18-year legal threshold is methodologically limited and not forensically robust. A multidisciplinary approach is instead strongly recommended, combining multiple anatomical sites for age estimation, particularly teeth (third molar development), which continue their maturation beyond 18 years of age. Dental age estimation is supported by a substantial body of peer-reviewed literature demonstrating validated statistical performance, quantified error rates, and accuracy metrics specifically calibrated for the 18-year legal threshold discrimination.

## Figures and Tables

**Table 1:** Predefined categories for data extraction according to the standardized form based on PRISMA-DTA recommendations [27].

Study Characteristics	Population Characteristics	Index Test Methods	Reference Standard	Overall Accuracy Outcomes	Diagnostic Performance (Threshold- Specific)	Age-Specific and Subgroup Results	Statistical Methods
Author, publication year, country, language	Total sample size, age range	Method used (GP, TW2, TW3, AI/ML, epiphyseal fusion, other)	Source of chronological age veification	MAE with SD or 95% CI	Age threshold examined (16, 18, 21 years)	Results stratified by sex	Statistical tests used
Study design	Sex distribution	Imaging modality (plain radiography specifications)	Verification method and documentation	Mean Squared Error (MSE) with SD or 95% CI	Sensitivity and Specificity with 95% CI	Results stratified by age categories	Significance levels and *p*-values
Setting context	Ethnicity/race (as reported by authors)	Anatomical site	Blinding of reference standard to index test	Root Mean Square Error (RMSE)	Positive Predictive Value (PPV) and Negative Predictive Value (NPV) with 95% CI	Results stratified by ethnicity or geographic origin	Confidence interval methods
Recruitment period, sample size calculation	Geographic origin, nationality	Number of raters, rater qualifications and training	Time interval between index test and reference standard	Correlation coefficients (Pearson r, Spearman ρ) with 95% CI	Accuracy (proportion correctly classified) with 95% CI	Results stratified by method or rater	Handling of missing data
Funding sources, conflicts of interest	Inclusion and exclusion criteria	Blinding of the evaluation		Concordance correlation coefficient	Area Under Receiver Operating Characteristic Curve (AUC/AUROC) with 95% CI	Interaction effects	Sample size and power calculations
Ethics approval and informed consent	Health status, medical conditions	Interpretation protocol and decision rules		Limits of agreement (Bland-Altman)	Diagnostic Odds Ratio (DOR) with 95% CI		Software used for analysis
	Recruitment method	Software or tools used		R^2^ or coefficient of determination	Positive Likelihood Ratio (LR+) with 95% CI		
		Time required for assessment			Negative Likelihood Ratio (LR−) with 95% CI		

**Table 2:** Included 23 studies’ characteristics. Legend: GP = Greulich–Pyle atlas; LR = Linear regression; TN = Thiemann and Nitz atlas; TW2 and TW3 = Tanner-Whitehouse 2 and 3; KG Atlas = GÖK atlas; GR Atlas = Gilsanz-Ratib altas, AI = Artificial Intelligence; DL = Deep Learning; ML = Machine Learning; MAE = Mean Absolute Error; ID = Identity Document; Yes-1 = the study has been included in meta-analyses 1; Yes-2 = the study has been included in meta-analyses 2.

*Study*	*Characteristics*							*Outcomes*					*Meta-Analysis Inclusion*								
	Country	Study Design	Context	Sample (*n*)	Age (Yrs)	Male (%)	Methods	Overall Accuracy					Diagnostic Performance at the 18 Years Age Threshold								
								*Inter-Rater Reliability*	*MAE ± SD (Years)*		*MSE ± SD (Years)*	*RMSE (Years)*	*Sensitivity* *(%)*	*Specificity* *(%)*	*PPV (%)*	*NPV (%)*	*Accuracy (%)*	*AUC (95%CI)*	*LR+*	*LR−*	
*Pinchi* et al., *2026* [35]	Italy	Retrospective study	Clinical	453	6–20	50	GP	/	0.81 ± 0.73		/	/	/	/	/	/	/	/	/	/	Yes-2
							TW3	/	0.70 ± 0.64		/	/	/	/	/	/	/	/	/	/	
							ML-based	/	0.62 ± 0.51		/	/	/	/	/	/	/	/	/	/	
*Pereira* et al., *2025* [36]	Portugal	Cross-sectional	Clinical	597	6–21	49.6	GP	κ values 0.965–0.993	M = 1.88		/	/	96.98	64	/	/	91.46	0.950	/	/	Yes-1
									F = 1.39		/	/	/	/	/	/	/	/	/	/	
									Overall = 1.63		/	/	/	/	/	/	/	/	/	/	
							LR on GP-16 bones	κ values 0.965–0.993	M = 1.47		/	/	95.37	50.63	/	/	89.45	0.908	/	/	
									F = 1.53		/	/	/	/	/	/	/	/	/	/	
									Overall = 1.51		/	/	/	/	/	/	/	/	/	/	
*Wang* et al., *2025* [37]	China	Retrospective study	Clinical	688	11–24	52.1	TW3	K values 0.81–0.74	/		/	/	Expert 1 = 36	Expert 1 = 83	Expert 1 = 50	Expert 1 = 74	Expert 1 = 71	/	/	/	Yes-1
									/		/	/	Expert 2 = 46	Expert 2 = 97	Expert 2 = 50	Expert 2 = 94	Expert 2 = 93	/	/	/	
									/		/	/	Expert 3 = 39	Expert 3 = 89	Expert 3 = 55	Expert 3 = 82	Expert 3 = 79	/	/	/	
									/		/	/	Expert 4 = 43	Expert 4 = 92	Expert 4 = 50	Expert 4 = 88	Expert 4 = 86	/	/	/	
							Automated TW3 CNN-based		/		/	/	CNN1 = 70	CNN1 = 80	CNN1 = 63	CNN1 = 77	CNN1 = 77	/	/	/	
									/		/	/	CNN2 = 68	CNN2 = 92	CNN2 = 65	CNN2 = 90	CNN2 = 88	/	/	/	
									/		/	/	CNN3 = 68	CNN3 = 93	CNN3 = 68	CNN3 = 91	CNN3 = 89	/	/	/	
									/		/	/	CNN4 = 100	CNN4 = 100	CNN4 = 100	CNN4 = 100	CNN4 = 100	/	/	/	
*Malkoçoğlu* et al., *2025* [38]	Turkey	Retrospective study	Forensic	700	12–18	50	KG Atlas	ICC Females 0.404 ICC Males 0.878	/		/	/	/	/	/	/	/	/	/	/	No
							GR Atlas		/		/	/	/	/	/	/	/	/	/	/	
									/		/	/	/	/	/	/	/	/	/	/	
									/		/	/	/	/	/	/	/	/	/	/	
*Behera* et al., *2023* [39]	India	Cross-sectional	Forensic	134	8–20	47.7	GP	K value 0.72	/		/	/	/	/	/	/	/	/	/	/	No
*Santoro* et al., *2019* [40]	Italy, Africa	Cross-sectional	Clinical	204	4–19	50.0	GP	/	/		/	/	/	/	/	/	/	/	/	/	No
							TW2	/	/		/	/	/	/	/	/	/	/	/	/	
							FELS	/	/		/	/	/	/	/	/	/	/	/	/	
*Maggio* et al., *2018* [41]	Australia	Cross-sectional	Clinical	360	<25	50	GP	K value 0.887	/		SD only	/	/	/	/	/	SD only	/	/	/	No
											M ±0.90						M ±0.00–3.53				
											F ±0.45						F ±0.28–5.58				
*Alcina* et al., *2018* [42]	Spain	Cross-sectional	Clinical	1150	M < 19 F < 18	51.3	GP	ρc value 0.99	/		/	/	2 categories	2 categories	2 categories	2 categories	/	/	/	/	Yes-1
													> 18 > 19	> 18 > 19	> 18 > 19	> 18 > 19					
													97 (89–99)	72 (59–81)	77 (67–85)	95-					
									/		/	/	3 categories	3 categories	3 categories	3 categories	/	/	/	/	
													> 16 > 17 > 18	> 16 > 17 > 18	> 16 > 17 > 18	> 16 > 17 > 18					
													96 (86–99)	100 (92–100)	100 (92–100)	95-					
*Thodberg* et al., *2017* [43]	Switzerland, Denmark, Netherlands, USA	Retrospective study	Clinical	2038	<21		AI BoneXpert GP-based	/	/		/	Range only	70	90	87	75	80		10	30	Yes-1
												M 0.45–0.78									
												F 0.52–0.75									
												Overall 0.54–0.73									
*Kumar* et al., *2016* [44]	India	Cross-sectional	Forensic	120	15–19	50	Epiphyseal fusion	/	/		/	/	/	/	/	/	/	/	/	/	No
*Maggio* et al., *2016* [45]	Australia	Cross-sectional	Clinical	360	<25	50	TW2	K values 0.869–1	/		/	/	/	/	/	/	/	/	/	/	No
							TW3														
*Gelbrich* et al., *2015* [46]	Germany	Retrospective study	Clinical	382	7.8–19	44	TN Atlas	/	/		/	/	/	/	/	/	/	/	/	/	No
*Mohammed* et al., *2015* [47]	India	Cross-sectional	Forensic	660	9–20	50	GP	ICC 0.96	F = 1.42		/	/	/	/	/	/	/	/	/	/	No
									M = 1.14		/										
									Overall = 1.27		/										
*Pinchi* et al., *2014* [48]	Italy	Retrospective study	Clinical	307	6–20	52.7	GP	ICC 0.948	/		/	/	/	/	/	/	/	/	/	/	No
							TW2	ICC 0.931	/		/	/	/	/	/	/	/	/	/	/	
							TW3	ICC 0.929	/		/	/	/	/	/	/	/	/	/	/	
*De Donno* et al., *2013* [49]	Italy	Cross-sectional	Clinical	300	10–20	51.3	GP	F = 1.05	Expert 1		/	/	/	/	/	/	/	/	/	/	Yes-2
									0.27 ± 0.27												
									Expert 2		/	/	/	/	/	/	/	/	/	/	
									0.16 ± 1.46												
									Expert 3		/	/	/	/	/	/	/	/	/	/	
									0.09 ± 2.12												
									Expert 4		/	/	/	/	/	/	/	/	/	/	
									0.10 ± 2.07												
*Kadam* et al., *2012* [50]	India	Cross-sectional	Clinical	120	12–20	50	Epiphyseal fusion	/	/		/	/	/	/	/	/	/	/	/	/	No
*Hackman* et al., *2012* [51]	Scotland	Cross-sectional	Clinical	818	1–21	66.6	GP	/	/		/	/	/	/	/	/	/	/	/	/	No
*Tisè* et al., *2011* [52]	Italy	Retrospective study	Clinical	484	11–19	74.8	GP	K value 0.949	Expert 1 *		/	/	/	/	/	/	/	/	/	/	Yes-2
									M 0.66 ± 0.57												
									F 0.72 ± 0.56												
									Expert 2 *		/	/	/	/	/	/	/	/	/	/	
									M 0.69 ± 0.57												
									F 0.91 ± 0.68												
*Schmidt* et al., *2008* [53]	Germany	Cross-sectional	Forensic	649	1–18	53.3	GP	/	/	/		/	/	/	/	/	/	/	/	/	No
*Schmidt* et al., *2007* [54]	Germany	Cross-sectional	Forensic	649	1–18	53.3	GP	/	/	/		/	/	/	/	/	/	/	/	/	No
							TN Atlas					/	/	/	/	/	/	/	/	/	
*Schmeling* et al., *2006* [55]	Germany	Overview	Forensic		/	/	/	/	/	/		/	/	/	/	/	/	/	/	/	No
*Yarımoğlu* et al., *2005* [56]	Turkey	Cross-sectional	Clinical	138	0–19	66.6	GP	/	/	/		/	/	/	/	/	/	/	/	/	No
*Garamendi* et al., *2003* [57]	Moroccan pupolation	Cross-sectional	Forensic	114	13–25	100	GP	ICC 0.93	/	/		/	Age range	Age range	Age range	Age range	/	0.77	Age range	Age range	Yes-1
													17/18 = 68	17/18 = 78	17/18 = 79	17/18 = 66			17/18 = 3.19	17/18 = 0.41	
													18/19 = 45	18/19 = 86	18/19 = 80	18/19 = 56			18/19 = 3.29	18/19 = 0.64	

* The original study reported the standard error (SE), creating conflicts in meta-analyses tests. We converted the SE in SD according to the formula SD = SE × √*N*. Results: Expert 1 Males: SD = 0.57 (was 0.03), *N* = 357/Expert 1 Females: SD = 0.56 (was 0.05), *N* = 127/Expert 2 Males: SD = 0.57 (was 0.03), *N* = 357/Expert 2 Females: SD = 0.68 (was 0.06), *N* = 127.

**Table 3:** Pooled diagnostic accuracy by assessment method. Note: The CNN subgroup results derive entirely from a single study (Wang et al. 2025) [37] conducted on 688 Chinese clinical participants. These results are not directly comparable to the GP or TW3 subgroups, which aggregate multiple independent studies and populations. The CNN estimates should be interpreted as preliminary, single-study findings requiring independent external validation in forensic populations before any conclusions about the superiority of AI-based approaches can be drawn.

Method	Sensitivity	Specificity	Accuracy	*N* Values	*N* Participants
**CNN**	76.5%	91.3%	88.6%	4	688
**GP/GP-based**	82.6%	77.3%	86.9%	7	4496
**TW3**	41.0%	90.3%	82.3%	4	688

**Table 4:** Pooled diagnostic accuracy by population type. pp = percentage points.

Outcome	Clinical (*n* = 11)	Forensic (*n* = 2)	Difference
**Sensitivity**	71.3% (63.1–79.6%)	56.6% (34.0–79.1%)	**+14.8 pp**
**Specificity**	85.9% (83.8–88.0%)	82.3% (74.5–90.1%)	**+3.6 pp**
**Accuracy**	85.9% (79.4–92.3%)	/	

**Table 5:** Sensitivity test results of comparison between pooled measures of overall analysis and low/high-risk studies.

	Overall	Low Risk Only	Difference Overall-Low Risk	High Risk Only	Difference Overall-High Risk
**Sensitivity**	69.5%	64.8%	**+4.6 pp**	87.5%	**−18 pp**
	(61.7–77.3%)	(55.1–74.6%)		(71.88–100%)	
**Specificity**	85.6%	89.9%	**−4.3 pp**	68.3%	**+17.3 pp**
	(83.5–87.6%)	(88.2–91.6%)		(42.4–94.2%)	
**Accuracy**	85.9%	85.4%	**+0.4 pp**	86.9%	**−1.1 pp**
	(79.4–92.3%)	(77.8–93.1%)		(79.4–94.5%)	

**Table 6:** Pooled MAE by assessment method.

Method	MAE (Years)	95% CI	*N* Estimates	*N* Participants
ML-based	0.620	0.573–0.667	1	453
TW3	0.700	0.641–0.759	1	453
GP	0.503	0.311–0.695	9	2494

**Table 7:** Univariate meta-regression results of MAE according to population type (forensic vs. clinical application), method type (GP vs. TW3/ML-based), sample size (log-transformed), study (Pinchi 2026 vs. De Donno/Tisè), and sex (males vs. females—Tisè only).

Factor	R^2^ (Variance Explained)	Effect Size	*p*-Value	Rank
Population Type	29.1%	Forensic +0.47 years	<0.001	#1
Method Type	12.1%	GP +0.16 years	<0.001	#2
Sample Size	4.0%	−0.21 per log-unit	<0.001	#3
Sex (Tisè only)	96.3%	Males −0.12 years	0.006	#1
Full Model	36.8%	All 3 combined	<0.001	–

**Table 8:** Summary of key meta-analytic findings.

Included Studies	Outcome	Pooled Estimate	95% CI	Heterogeneity (I^2^)	Prediction Interval (PI) for Future Studies	GRADE Certainty
**5 [36,37,42,43,57]**	Pooled Sensitivity (18-yr threshold)	69.5%	61.7–77.3%	99.8%	35.3–100%	**VERY LOW**
**5 [36,37,42,43,57]**	Pooled Specificity (18-yr threshold)	85.6%	83.5–87.6%	99.4%	77.2–93.9%	**VERY LOW**
**5 [36,37,42,43,57]**	Overall Accuracy	85.9%	79.4–92.3%	99.4%	60.3–100%	**VERY LOW**
**5 [36,37,42,43,57]**	Pooled AUC (SROC)	0.775	/	/		**VERY LOW**
**3 [35,49,52]**	**Pooled MAE (all methods)**	**0.537 years**	**0.387–0.686 years**	**98.1%**	**0.33–0.79 years**	**VERY LOW**

## Data Availability

The original contributions presented in this study are included in the article/Supplementary Material. Further inquiries can be directed to the corresponding author.

## Supplementary Materials

The following supporting information can be downloaded at: https://www.mdpi.com/article/10.3390/diagnostics16132093/s1, Appendix S1: Complete Search Strategies; Appendix S2: PRISMA 2020 Checklist; Appendix S3: Excluded Studies with Reasons; Appendix S4: Data Extraction Forms.
