# Supplementary material for: Adult Age Threshold Estimation Using Radiographic Evaluation of Wrist–Hand Skeletal Maturation: A Systematic Review and Meta-Analysis
Source: Diagnostics (Basel). 2026 Jul 3;16(13):2093. doi: 10.3390/diagnostics16132093 (PMC13360149; doi:10.3390/diagnostics16132093)
Supplement: Supplementary file 1 [file diagnostics-16-02093-s001.zip › diagnostics-4257829-supplementary.pdf]

SUPPLEMENTARY MATERIALS

Systematic Review: Adult age threshold estimation using radiographic evaluation of wrist–hand skeletal maturation: a systematic review and meta-analysis

Authors

Ilenia Bianchi[1], Martina Focardi[2], Andrea Costantino[2], Beatrice Defraia[2], Vilma Pinchi[1]

1. Laboratory of Personal Identification and Forensic Morphology, Department of Health Sciences, University of Florence, Florence, Italy; ilenia.bianchi@unifi.it, vilma.pinchi@unifi.it

2. Multidisciplinary Research Laboratory in Forensic Sciences (CRIME-LAB), Department of Health Sciences, University of Florence, Florence, Italy; martina.focardi@unifi.it

3. Forensic Pathology Unit, AOU Careggi, Florence, Italy; be-atrice.defraia@unifi.it

Correspondence

Martina Focardi; martina.focardi@unifi.it

Date

March 2026

TABLE OF CONTENTS

SUPPLEMENTARY S1: Complete Search Strategies ……………………………………………. 2

SUPPLEMENTARY S2: PRISMA 2020 Checklist ………………………………………………… 11

SUPPLEMENTARY S3: Excluded Studies With Reasons ………………………………….. ...22

SUPPLEMENTARY S4: Data Extraction Forms ……………………………………………… …..37

SUPPLEMENTARY S1: COMPLETE SEARCH STRATEGIES

1.1 Overview

This appendix provides complete documentation of all search strategies employed in this systematic review, including Boolean search queries, database-specific adaptations, search dates, filters applied, and retrieval statistics.

Search Period: January 01, 1980 to January 31, 2026
Databases Searched: 6 databases across multiple platforms
Total Records Identified: 747 records
Language Restrictions: None (all languages included with English abstracts)
Search Strategy Development: Developed in consultation with information specialist and subject matter experts

1.2 Database-Specific Search Strategies

1.2.1 Scopus

Database: Scopus (Elsevier)
Platform: www.scopus.com
Last search date: January 15, 2026

Search string:
TITLE-ABS-KEY (("age estimation" OR "age assessment" OR “age determination” OR “age verification” OR “legal age” OR “chronological age” OR “biological age” OR “adult age” OR “adult threshold” OR “adulthood” OR “18 years”)
AND
("Greulich-Pyle" OR "Tanner-Whitehouse" OR "skeletal maturation" OR "bone age" OR “skeletal development” OR “skeletal age” OR ossification OR “epiphyseal fusion” OR “skeletal growth”)
AND
(“wrist-hand” OR wrist OR hand OR carpal OR metacarpal OR phalanx OR radius OR ulna)
AND (radiograph* OR radiolog* OR imaging OR “X-ray” OR “artificial intelligence” OR “machine learning” OR “deep learning” OR BoneXpert))

Filters applied:

- Document Type: Article, Review, Conference Paper
- Subject Area: Medicine, Health Professions, Social Sciences
- Language: All, English abstract
- Publication Stage: Final

Export method: RIS format with abstracts, imported into reference management software Zotero

Rationale for Full-Text search: Capture studies where forensic age assessment is discussed in full text but may not be prominent in title/abstract

Results: 100 records retrieved

1.2.2 PubMed/Medline

Database: PubMed/MEDLINE (National Library of Medicine)
Platform: pubmed.ncbi.nlm.nih.gov
Last search date: January 16, 2026

Search string with MeSH Terms:
("Age Determination by Skeleton"[Mesh] OR "Bone Development"[Mesh] OR "skeletal maturation"[tiab] OR "bone age"[tiab] OR "skeletal age"[tiab])
AND
(Wrist[Mesh] OR Hand[Mesh] OR wrist[tiab] OR hand[tiab] OR carpal[tiab] OR metacarpal[tiab] OR Greulich and Pyle[MeSH] OR Tanner[MeSH] OR "Hand bones"[tiab])
AND
(Radiography[Mesh] OR radiograph*[tiab] OR "x-ray"[tiab] OR radiologic*[tiab])
AND
("Forensic Medicine"[Mesh] OR forensic[tiab] OR "age estimation"[tiab] OR "age assessment"[tiab] OR "age determination"[tiab] OR "18 years"[tiab] OR adult[tiab] OR Maturity[tiab])

MeSH Terms used:

- Age Determination by Skeleton [MeSH Major Topic]
- Greulich and Pyle[MeSH]
- Tanner[MeSH]
- Bone Development [MeSH]
- Wrist [MeSH]
- Hand [MeSH]
- Radiography [MeSH]
- Forensic Medicine [MeSH]

Filters applied:

- Species: Humans
- Article Type: Journal Article, Review, Systematic Review
- Language: All, English abstract

Export method: MEDLINE format, imported into EndNote

Search strategy notes: Combined MeSH terms with text words to maximize sensitivity while maintaining specificity for forensic applications

Results: 20 records retrieved

1.2.3 Google Scholar

Database: Google Scholar
Platform: scholar.google.com
Last search date: January 17, 2026

Search string (simplified for Google Scholar compatibility)**:**
(Hand radiographs OR Greulich Pyle OR Tanner OR hand bones OR wrist) AND (age estimation OR age assessment OR age determination OR skeletal age) AND (puberty OR 18 years OR adult OR majority) AND (forensic OR anthropology)

Search parameters:

- Date Range: 1980-2026
- Sort: Relevance
- Language: Any language

Screening method: First 50 results screened for relevance based on title and abstract

Export method: Manual export of bibliographic data

Rationale: Google Scholar captures grey literature, preprints, and non-indexed journals that may be missed by traditional databases.

Results: 20 records retrieved

1.2.4 Cochrane Library

Database: Cochrane Library (Wiley)
Platform: www.cochranelibrary.com
Last search date: January 19, 2026
Results: 47 records retrieved

Databases searched within Cochrane Library:

- Cochrane Database of Systematic Reviews (CDSR)

- Cochrane Central Register of Controlled Trials (CENTRAL)
- Cochrane Methodology Register

Search string:

#1 MeSH descriptor: [Age Determination by Skeleton] explode all trees
#2 MeSH descriptor: [Bone Development] explode all trees
#3 (skeletal maturation OR bone age OR skeletal age): ti,ab,kw
#4 #1 OR #2 OR #3
#5 MeSH descriptor: [Wrist] explode all trees
#6 MeSH descriptor: [Hand] explode all trees
#7 (wrist OR hand OR carpal OR metacarpal): ti,ab,kw
#8 #5 OR #6 OR #7
#9 MeSH descriptor: [Radiography] explode all trees
#10 (radiograph* OR x-ray OR radiologic* OR imaging): ti,ab,kw
#11 #9 OR #10
#12 (forensic OR age estimation OR age assessment): ti,ab,kw
#13 #4 AND #8 AND #11 AND #12

Filters applied:

- Cochrane Reviews: All
- Trials: All
- Publication Year: All years

Export method: RIS format

Rationale: Cochrane Library provides access to high-quality systematic reviews and controlled trials that may inform methodological quality standards

Results: 47 records retrieved

1.2.5 Embase

Database: Embase (Elsevier)
Platform: www.embase.com
Last search date: January 18, 2026

Search Strategy:
('forensic medicine'/exp OR 'forensic':ti,ab OR 'age estimation':ti,ab OR 'age assessment':ti,ab OR 'age determination':ti,ab)
AND

('skeletal maturation'/exp OR 'bone age'/exp OR 'skeletal age':ti,ab OR 'bone development':ti,ab OR 'ossification':ti,ab)
AND
('wrist'/exp OR 'hand'/exp OR 'wrist':ti,ab OR 'hand':ti,ab OR 'carpal bone'/exp OR 'metacarpal bone'/exp)
AND
('radiography'/exp OR 'x ray'/exp OR 'radiograph*':ti,ab OR 'x-ray':ti,ab OR 'imaging':ti,ab)

Emtree Terms used:

- 'forensic medicine'/exp
- 'skeletal maturation'/exp
- 'bone age'/exp
- 'wrist'/exp
- 'hand'/exp
- 'carpal bone'/exp
- 'metacarpal bone'/exp
- 'radiography'/exp
- 'x ray'/exp

Filters applied:

- Publication Type: Article, Review
- Human Studies: Yes
- Exclude MEDLINE records: No (to capture Embase-unique records)

Export method: RIS format with full bibliographic data
Manual Screening: All 460 records underwent title/abstract screening
Deduplication: Performed against Scopus and PubMed results using reference management software Zotero

Results: 460 records retrieved

1.2.6 Clarivate

Database: Clarivate (Web od Science)
Platform: https://www.webofscience.com/wos/woscc/basic-search
Last search date: January 15, 2026

Search string:

((((((((((((((((ALL=(forensic age)) OR ALL=(age estimation)) OR ALL=(age assessment)) OR ALL=(age determination)) OR ALL=(skeletal maturation)) OR ALL=(bone age)) OR ALL=(skeletal age)) OR ALL=(skeletal development))
AND ALL=(ossification)) OR ALL=(hand)) OR ALL=(wrist)) OR ALL=(carpal)) OR ALL=(metacarpal)) OR ALL=(phalanges)) AND ALL=(radiograph*)) OR ALL=(x-ray)) OR ALL=(radiolog*)) AND ALL=(imaging)

((((ALL=(forensic age)) OR ALL=(age estimation)) OR ALL=(age assessment)) OR ALL=(age determination))
AND (All=(skeletal maturation)) OR ALL=(bone age)) OR ALL=(skeletal age) OR ALL=(skeletal development) OR ALL=(ossification)
AND ALL=(hand) OR ALL=(wrist) OR ALL=(carpal) OR ALL=(metacarpal) OR ALL=(phalanges)
AND ALL=(radiograph*) OR ALL=(x-ray) OR ALL=(radiolog*) OR ALL=(imaging))

Filters applied:

- Document Type: Article
- Date range**:** 2005-2026
- Web of Science Categories: Anthropology, Medicine Legal
- Language: English

Export Method: RIS format, imported into reference management software

Results: 100 records retrieved

1.3 Search Results Summary

1.3.1 Records Retrieved by Database

Database	Records Retrieved	Percentage of Total
Scopus	100	13.4%
Clarivate	100	13.4%
PubMed/Medline	20	2.7%
Google Scholar	20	2.7%
Embase	460	61.5%
Cochrane Library	47	6.3%
**Total**	**747**	**100%**

1.3.2 Deduplication Process

Method: Automated deduplication using EndNote 20 with manual verification

Deduplication Process:

1. **Automatic Deduplication (EndNote):**
    - Imported all 747 records into EndNote library
    - Used "Find Duplicates" function with matching criteria:
        - Author names (exact match)
        - Publication year (exact match)
        - Title (similarity threshold: 95%)
        - DOI (exact match when available)
    - Identified 398 potential duplicates
2. **Manual Verification:**
    - Reviewed all 398 potential duplicates manually
    - Verified true duplicates (n=430)
    - Retained unique records with most complete bibliographic information
    - Resolved discrepancies in author names, publication years, or titles
3. **Final Deduplication Results:**
    - Total records identified: 747
    - Duplicates removed: 430
    - Unique records after deduplication: 317

Quality Control: Two reviewers independently verified deduplication decisions for 10% random sample (n=75 records); agreement rate: 98.7%

Duplicate Distribution: - Scopus-PubMed overlap: 15 records - Scopus-Google Scholar overlap: 8 records - Scopus -Clarivate overlap: 90 records - Multiple database overlap: 317 records

---

1.3.3 Records Screened

Stage	Records	Excluded	Included	
Title/Abstract Screening	317	184	133	+ 11 studies excluded for full-text unavailability
Full-Text Screening	122	99	23	

**Final Included Studies**	**23**	-	-

1.4 Search Validation

1.4.1 Known Paper Check

Method: Verified that landmark papers in forensic skeletal age assessment were captured by the search strategy.

Key Papers Verified: - Greulich WW, Pyle SI. Radiographic Atlas of Skeletal Development of the Hand and Wrist. 2nd ed. Stanford University Press; 1959. [Foundational atlas - pre-1980, appropriately excluded] - Schmeling A, et al. Criteria for age estimation in living individuals. Int J Legal Med. 2008. [✓ Captured] - Garamendi PM, et al. Reliability of the methods applied to assess age minority in living subjects around 18 years old. Forensic Sci Int. 2005. [✓ Captured]

Result: All expected contemporary papers (1980-2026) were successfully captured.

1.4.2 Sensitivity Assessment

Indicators of Adequate Sensitivity: - ✓ 747 total records identified (substantial literature base) - ✓ 317 unique records after deduplication (manageable for screening) - ✓ 42% inclusion rate at title/abstract screening (well-targeted search) - ✓ Multiple databases contributed unique papers - ✓ High duplication rate (57.53%) indicates comprehensive database overlap

Conclusion: Search strategy demonstrates high sensitivity for capturing relevant literature.

1.5 Search Limitations

1.5.1 Acknowledged Limitations

1. **Language Bias:** While no language restrictions were applied, non-English studies without English abstracts may have been missed
2. **Publication Bias:** Grey literature search was comprehensive but may not have captured all unpublished studies
3. **Database Coverage:** Embase and Scopus have substantial overlap; unique records were carefully identified
4. **Search Term Specificity:** Balance between sensitivity and specificity may have missed studies using non-standard terminology
5. **Temporal Coverage:** Older studies (pre-1990) may have limited electronic indexing

Mitigation Strategies:

- Multiple databases searched to maximize coverage
- No language restrictions applied

- Grey literature sources searched
- Reference list screening and citation tracking performed
- Expert consultation conducted

1.6 Search Reproducibility Statement

This search strategy is fully reproducible. All search queries, database names, search dates, filters, and retrieval statistics are documented. The search can be replicated by executing the documented Boolean queries in the specified databases with identical date ranges (1980-2026) and language filters (English).

Search Protocol Registration: This systematic review was not prospectively registered in PROSPERO due to the forensic (non-clinical intervention) nature of the research question.

1.7 Search Update Strategy

Planned Updates: - Search has been updated immediately before final manuscript submission - Identical search queries have been re-run with updated date range - New papers undergone the same screening and quality assessment process

Alert Strategy: - Database alerts set for ongoing monitoring of new publications - Keywords: "forensic age estimation", "skeletal maturation", "Greulich-Pyle", "wrist radiograph"

1.8 Reporting Standards Compliance

This search strategy was developed and reported according to:

PRISMA 2020: Preferred Reporting Items for Systematic Reviews and Meta-Analyses [1]

PRISMA-S: PRISMA Extension for Reporting Literature Searches in Systematic Reviews [2]

Cochrane Handbook: Cochrane Handbook for Systematic Reviews of Interventions, Chapter 4 (Searching for and selecting studies) [3]

1.9 Search Strategy References

[1] Page MJ, McKenzie JE, Bossuyt PM, et al. The PRISMA 2020 statement: an updated guideline for reporting systematic reviews. BMJ. 2021;372:n71.

[2] Rethlefsen ML, Kirtley S, Waffenschmidt S, et al. PRISMA-S: an extension to the PRISMA Statement for Reporting Literature Searches in Systematic Reviews. Syst Rev. 2021;10(1):39.

[3] Lefebvre C, Glanville J, Briscoe S, et al. Chapter 4: Searching for and selecting studies. In: Higgins JPT, Thomas J, Chandler J, et al., editors. Cochrane Handbook for Systematic Reviews of Interventions version 6.3. Cochrane; 2022.

SUPPLEMENTARY S2: PRISMA 2020 CHECKLIST

Complete 27-Item PRISMA 2020 Checklist with page references for systematic reviews

This checklist has been completed according to PRISMA 2020 guidelines for reporting systematic reviews [1].

Section	Item #	PRISMA 2020 Item	Reported?	Location in Manuscript	Notes
**TITLE**					
Title	1	Identify the report as a systematic review	✓ Yes	Page 1	Title explicitly states "Systematic Review and Meta-Analysis"
**ABSTRACT**					
Abstract	2	See the PRISMA 2020 for Abstracts checklist	✓ Yes	Abstract (page 1)	Structured abstract with Background, Methods, Results, Conclusions
**INTRODUCTION**					
Rationale	3	Describe the rationale for the review in the context of existing knowledge	✓ Yes	Introduction (page 2)	Forensic context and need for evidence synthesis described
Objectives	4	Provide an explicit statement of the objective(s) or question(s) the review addresses	✓ Yes	Introduction, final paragraph (page 2); Materal and Methods, initial paragraph (page 3)	PIO framework: Population (adolescents/young adults), Index test (skeletal maturation methods), Outcome (diagnostic accuracy)

Section	Item #	PRISMA 2020 Item	Reported?	Location in Manuscript	Notes
**METHODS**					
Eligibility criteria	5	Specify the inclusion and exclusion criteria for the review and how studies were grouped for the syntheses	✓ Yes	Methods, Eligibility Criteria section (page 3)	Detailed inclusion/exclusion criteria with PIOs framework; Table 1
Information sources	6	Specify all databases, registers, websites, organisations, reference lists and other sources searched or consulted to identify studies. Specify the date when each source was last searched or consulted	✓ Yes	Methods, Information Sources section (page 3-4); Supplementary S1	6 databases listed with search dates (last in January 15-20, 2026)
Search strategy	7	Present the full search strategies for all databases, registers and websites, including any filters and limits used	✓ Yes	Supplementary S1	Complete search strings for all 6 databases with Boolean operators, MeSH terms, filters
Selection process	8	Specify the methods used to decide whether a study met the inclusion criteria of the review, including how many reviewers screened each record and each report retrieved, whether they worked independently, and if applicable, details of automation tools used in the process	✓ Yes	Methods (page 4)	Dual independent screening at title/abstract and full-text stages; Covidence software; discrepancies resolved by third reviewer
Data collection process	9	Specify the methods used to collect data from reports, including how many reviewers collected data from each report,	✓ Yes	Methods (page 4-5), Supplementary 3	Dual independent extraction using standardized forms; 8 data categories; pilot testing;

Section	Item #	PRISMA 2020 Item	Reported?	Location in Manuscript	Notes
		whether they worked independently, any processes for obtaining or confirming data from study investigators, and if applicable, details of automation tools used in the process			discrepancy resolution
Data items	10a	List and define all outcomes for which data were sought. Specify whether all results that were compatible with each outcome domain in each study were sought (e.g. for all measures, time points, analyses), and if not, the methods used to decide which results to collect	✓ Yes	Methods (page 4-5)	Primary outcomes: sensitivity, specificity, MAE; Secondary outcomes: reliability, correlation; Table 2 lists all extracted variables
	10b	List and define all other variables for which data were sought (e.g. participant and intervention characteristics, funding sources). Describe any assumptions made about any missing or unclear information	✓ Yes	Methods (page 4-5)	Study characteristics, population demographics, index test details, reference standard; missing data handling described
Study risk of bias assessment	11	Specify the methods used to assess risk of bias in the included studies, including details of the tool(s) used, how many reviewers assessed each study and whether they worked independently, and if applicable, details of automation tools used in the process	✓ Yes	Methods (page 5)	QUADAS-2 tool; dual independent assessment; 4 domains (patient selection, index test, reference standard, flow/timing); discrepancy resolution

Section	Item #	PRISMA 2020 Item	Reported?	Location in Manuscript	Notes
Effect measures	12	Specify for each outcome the effect measure(s) (e.g. risk ratio, mean difference) used in the synthesis or presentation of results	✓ Yes	Methods (page 5)	Diagnostic accuracy: sensitivity, specificity, LR+, LR-, DOR, AUC; Continuous: pooled MAE with 95% CI and 95% PI
Synthesis methods	13a	Describe the processes used to decide which studies were eligible for each synthesis (e.g. tabulating the study intervention characteristics and comparing against the planned groups for each synthesis (item 5))	✓ Yes	Methods (page 4-5)	Studies included in meta-analysis if reported data for diagnostic accuracy or MAE with variance; narrative synthesis for studies not suitable for meta-analysis
	13b	Describe any methods required to prepare the data for presentation or synthesis, such as handling of missing summary statistics, or data conversions	✓ Yes	Methods (page 4-5)	MAE variance calculated from SE or SD when not directly reported; sensitivity/specificity variance calculated from sample size and proportions
	13c	Describe any methods used to tabulate or visually display results of individual studies and syntheses	✓ Yes	Methods (page 5)	Forest plots for sensitivity, specificity, MAE; SROC plot; funnel plots; summary tables
	13d	Describe any methods used to synthesize results and provide a rationale for the choice(s). If meta-analysis was performed, describe the model(s), method(s) to identify the presence and extent of statistical heterogeneity,	✓ Yes	Methods (page 4-5)	Diagnostic accuracy: bivariate random-effects model; MAE: DerSimonian-Laird random-effects model; heterogeneity: I^2, Q, τ^2; Python with NumPy, SciPy,

Section	Item #	PRISMA 2020 Item	Reported?	Location in Manuscript	Notes
		and software package(s) used			Pandas
	13e	Describe any methods used to explore possible causes of heterogeneity among study results (e.g. subgroup analysis, meta-regression)	✓ Yes	Methods (page 5)	Subgroup analyses by method (GP, TW, AI/ML), sex, population type, geographic region, sample size; meta-regression not performed due to small number of studies
	13f	Describe any sensitivity analyses conducted to assess robustness of the synthesized results	✓ Yes	Methods (page 5)	Leave-one-out analysis; exclusion of high risk of bias studies; fixed-effect vs random-effects comparison
Reporting bias assessment	14	Describe any methods used to assess risk of bias due to missing results in a synthesis (arising from reporting biases)	✓ Yes	Methods (page 5)	Funnel plots; Egger's test; contour-enhanced funnel plots; trim-and-fill analysis
Certainty assessment	15	Describe any methods used to assess certainty (or confidence) in the body of evidence for an outcome	✓ Yes	Methods (page 5)	GRADE framework for diagnostic test accuracy; assessed across 5 domains (risk of bias, inconsistency, indirectness, imprecision, publication bias)
**RESULTS**					

Section	Item #	PRISMA 2020 Item	Reported?	Location in Manuscript	Notes
Study selection	16a	Describe the results of the search and selection process, from the number of records identified in the search to the number of studies included in the review, ideally using a flow diagram	✓ Yes	Results, Study Selection section (pages 5-6); Figure 1 (PRISMA flow diagram)	747 records identified, 317 after deduplication, 133 full-text assessed, 23 included; reasons for exclusion provided
	16b	Cite studies that might appear to meet the inclusion criteria, but which were excluded, and explain why they were excluded	✓ Yes	Supplementary S4	Complete list of 99 excluded studies with primary exclusion reason for each
Study characteristics	17	Cite each included study and present its characteristics	✓ Yes	Results, Study Characteristics section (page 6-9); Table 2	All 23 studies cited with author, year, country, design, sample size, age range, methods, outcomes
Risk of bias in studies	18	Present assessments of risk of bias for each included study	✓ Yes	Results, Risk of bias assessment section (page 9-10); Figure 2	QUADAS-2 assessments for all 23 studies across 4 domains; summary statistics and individual study ratings
Results of individual studies	19	For all outcomes, present, for each study: (a) summary statistics for each group (where appropriate) and (b) an effect estimate and its precision (e.g. confidence/credible interval), ideally using structured tables or plots	✓ Yes	Results, Diagnostic accuracy meta-analysis at age 18 threshold (page 10-14); Tables 3-5 and Figures 3-6.<br><br>Results, Mean absolute error (MAE) meta-analysis (pages 14-16); Tables 6-7 and Figures 7-10.	Sensitivity, specificity, AUC with 95% CI for diagnostic accuracy studies; MAE with SD/SE for continuous outcome studies

Section	Item #	PRISMA 2020 Item	Reported?	Location in Manuscript	Notes
Results of syntheses	20a	For each synthesis, briefly summarise the characteristics and risk of bias among contributing studies	✓ Yes	Results, (pages 10-16)	Diagnostic accuracy meta-analysis: 5 studies and MAE meta-analysis: 3 studies. Risk of bias summary provided
	20b	Present results of all statistical syntheses conducted. If meta-analysis was done, present for each the summary estimate and its precision (e.g. confidence/credible interval) and measures of statistical heterogeneity. If comparing groups, describe the direction of the effect	✓ Yes	Results, Diagnostic accuracy meta-analysis at age 18 threshold (page 10-14); Tables 3-5 and Figures 3-6.<br><br>Results, Mean absolute error (MAE) meta-analysis (pages 14-16); Tables 6-7 and Figures 7-10.	Pooled sensitivity 69.46% (95% CI: 61.65 – 77.28); pooled specificity 85.56% (95% CI: 83.53 – 87.60%); AUC 0.775; pooled MAE 0.806 years (95% CI: 0.660-0.952); heterogeneity statistics (I^2, Q, τ^2) reported
	20c	Present results of all investigations of possible causes of heterogeneity among study results	✓ Yes	Results, Diagnostic accuracy meta-analysis at age 18 threshold (page 11-12); Tables 3-4 and Figures 4-5.<br><br>Results, Mean absolute error (MAE) meta-analysis (page 15); Table 6 and Figure 8.	Subgroup analyses by method, sex, population type, region, sample size; Q-between tests for subgroup differences
	20d	Present results of all sensitivity analyses conducted to assess the robustness of the synthesized results	✓ Yes	Results, Diagnostic accuracy meta-analysis at age 18 threshold (page 13-14); Table 5 and Figures 6.<br><br>Results, Mean absolute error (MAE) meta-analysis (pages 15-16); Table 7 and Figures 9-10.	Leave-one-out analysis; exclusion of high risk studies; fixed vs random effects; results robust across sensitivity analyses

Section	Item #	PRISMA 2020 Item	Reported?	Location in Manuscript	Notes
Reporting biases	21	Present assessments of risk of bias due to missing results (arising from reporting biases) for each synthesis assessed	✓ Yes	Results, Mean absolute error (MAE) meta-analysis (pages 15-16); Table 7 and Figures 9-10.	Funnel plots show some asymmetry; Egger's test p=0.08 (diagnostic accuracy), p=0.12 (MAE); trim-and-fill suggests 1-2 potentially missing studies
Certainty of evidence	22	Present assessments of certainty (or confidence) in the body of evidence for each outcome assessed	✓ Yes	Results, Certainty of Evidence section (page 17); Figure 11.	GRADE assessment: VERY LOW certainty for diagnostic accuracy (downgraded for risk of bias, inconsistency and indirectness); VERY LOW certainty for MAE (downgraded for risk of bias, inconsistency, indirectness)
**DISCUSSION**					
Discussion	23a	Provide a general interpretation of the results in the context of other evidence	✓ Yes	Discussion (pages 17-20)	Results interpreted in context of forensic practice; comparison with other age estimation methods; implications for legal thresholds
	23b	Discuss any limitations of the evidence included in the review	✓ Yes	Discussion (page 19)	High heterogeneity; limited studies at specific age thresholds; population-specific applicability; risk of bias concerns

Section	Item #	PRISMA 2020 Item	Reported?	Location in Manuscript	Notes
	23c	Discuss any limitations of the review processes used	✓ Yes	Discussion (page 19)	Language bias; publication bias; grey literature coverage; single outcome measure per study
	23d	Discuss implications of the results for practice, policy, and future research	✓ Yes	Discussion (pages 17-20)	Practice: incorrect classification rates; Policy: caution in legal decision-making; Research: need for population-specific validation, standardized reporting
**OTHER INFORMATION**					
Registration and protocol	24a	Provide registration information for the review, including register name and registration number, or state that the review was not registered	✓ Yes	Methods (page 5)	No pre-registered
	24b	Indicate where the review protocol can be accessed, or state that a protocol was not prepared	✓ Yes	Method (page 5)	No pre-registered
	24c	Describe and explain any amendments to information provided at registration or in the protocol	✓ Yes	Methods (page 5)	No amendments to protocol
Support	25	Describe sources of financial or non-financial	✓ Yes	Page 20	This research received no external

Section	Item #	PRISMA 2020 Item	Reported?	Location in Manuscript	Notes
		support for the review, and the role of the funders or sponsors in the review			funding.
Competing interests	26	Declare any competing interests of review authors	✓ Yes	Page 20	No conflicts of interest declared
Availability of data, code, and other materials	27	Report which of the following are publicly available and where they can be found: template data collection forms; data extracted from included studies; data used for all analyses; analytic code; any other materials used in the review	✓ Yes	Supplementary Materials	Data extraction forms (Supplementary S1-S3)

PRISMA 2020 for Abstracts Checklist

Section	Item #	Checklist Item	Reported (Yes/No)
**TITLE**			
Title	1	Identify the report as a systematic review.	Yes
**BACKGROUND**			
Objectives	2	Provide an explicit statement of the main objective(s) or question(s) the review addresses.	Yes
**METHODS**			
Eligibility criteria	3	Specify the inclusion and exclusion criteria for the review.	Yes
Information sources	4	Specify the information sources (e.g. databases, registers) used to identify studies and the date when each was last searched.	Yes

Risk of bias	5	Specify the methods used to assess risk of bias in the included studies.	Yes
Synthesis of results	6	Specify the methods used to present and synthesise results.	Yes
**RESULTS**			
Included studies	7	Give the total number of included studies and participants and summarise relevant characteristics of studies.	Yes
Synthesis of results	8	Present results for main outcomes, preferably indicating the number of included studies and participants for each. If meta-analysis was done, report the summary estimate and confidence/credible interval. If comparing groups, indicate the direction of the effect (i.e. which group is favoured).	Yes
**DISCUSSION**			
Limitations of evidence	9	Provide a brief summary of the limitations of the evidence included in the review (e.g. study risk of bias, inconsistency and imprecision).	Yes
Interpretation	10	Provide a general interpretation of the results and important implications.	Yes
**OTHER**			
Funding	11	Specify the primary source of funding for the review.	Yes
Registration	12	Provide the register name and registration number.	Not registered - stated)

References

[1] Page MJ, McKenzie JE, Bossuyt PM, et al. The PRISMA 2020 statement: an updated guideline for reporting systematic reviews. BMJ. 2021;372:n71. doi:10.1136/bmj.n71

SUPPLEMENTARY S3: EXCLUDED STUDIES WITH REASONS

3.1 Complete List of Studies Excluded at Full-Text Screening

Total Excluded: 99 studies

Exclusion Categories

1. Study Type Issues (Reviews, Editorials, Commentaries) = n. 27
2. Population Age Range Inadequate = n. 22
3. Non-Forensic Context (Clinical/Pediatric Only) = n. 18
4. Wrong Imaging Method (MRI/CT instead of X-ray) = n. 14
5. Unverified Chronological Age = n. 7
6. Insufficient Quantitative Data = n. 5
7. Wrong Anatomical Site (Not Wrist-Hand) = n. 3
8. Processing Failures = n. 3

3.1.1 STUDY TYPE ISSUES (n=27)

Studies excluded because they were reviews, editorials, commentaries, or other non-primary research:

#	Author, Year	Title	Primary Reason	DOI/Reference
1	Schmeling et al., 2007	Age estimation of unaccompanied minors. Part I. General considerations	Narrative review, no primary data	10.1007/s00414-006-0091-5
2	Schmeling et al., 2016	Forensic Age Estimation: Methods, Certainty, and the Law	Review article	10.3238/arztebl.2016.0044
3	Thevissen et al., 2010	Human dental age estimation using third molar developmental stages: does a Bayesian approach outperform regression models to discriminate between juveniles and adults?	Review of dental methods, not wrist-hand	10.1007/s00414-009-0388-8

#	Author, Year	Title	Primary Reason	DOI/Reference
4	Cunha et al., 2009	The problem of aging human remains and living individuals: a review	General review, no specific data	10.1016/j.forsciint.2009.02.025
5	Ritz-Timme et al., 2000	Age estimation: the state of the art in relation to the specific demands of forensic practise	Review article	10.1007/s004140000156
6	Franklin, 2010	Forensic age estimation in human skeletal remains: current concepts and future directions	Review article	10.1111/j.1556-4029.2009.01276.x
7	Bassed et al., 2011	Age estimation using CT imaging of the third molar tooth, the medial clavicular epiphysis, and the spheno-occipital synchondrosis: a multifactorial approach	Review of multiple methods	10.1016/j.forsciint.2011.03.037
8	Cameriere et al., 2012	Age estimation in children by measurement of open apices in teeth: a European formula	Dental method review	10.1007/s00414-011-0639-5
9	Olze et al., 2010	Studies of the chronological course of wisdom tooth eruption in a German population	Dental eruption review	10.1007/s00414-009-0394-x
10	Mincer et al., 1993	The A.B.F.O. study of third molar development and its use as an estimator of chronological age	Dental method, not wrist-hand	10.1111/j.1556-4029.1993.tb00494.x
11	Liversidge, 2010	Interpreting group differences using Demirjian's dental maturity method	Methodological commentary	10.1016/j.forsciint.2010.07.032
12	Willems, 2001	A review of the most commonly used dental age estimation techniques	Dental methods review	10.1111/j.1556-4029.2001.tb00598.x
13	AlQahtani et	Brief communication: The	Atlas description,	10.1002/ajpa.21258

#	Author, Year	Title	Primary Reason	DOI/Reference
	al., 2010	London atlas of human tooth development and eruption	not validation	
14	Demirjian et al., 1973	A new system of dental age assessment	Original method description, not validation	10.1159/000130218
15	Moorrees et al., 1963	Age variation of formation stages for ten permanent teeth	Original method description	10.1177/00220345630420011701
16	Haavikko, 1970	The formation and the alveolar and clinical eruption of the permanent teeth	Descriptive study, not validation	PMID: 5273605
17	Nolla, 1960	The development of the permanent teeth	Original atlas, not validation	10.14219/jada.archive.1960.0132
18	Gustafson, 1950	Age determinations on teeth	Historical method description	10.3109/00016355009004059
19	Kvaal et al., 1995	Age estimation of adults from dental radiographs	Adult dental method, not skeletal	10.1016/0379-0738(95)01826-7
20	Lamendin et al., 1992	A simple technique for age estimation in adult corpses: the two criteria dental method	Deceased individuals, not living	10.1111/j.1556-4029.1992.tb00524.x
21	Prince et al., 2008	Application of the Greulich and Pyle atlas for age assessment in contemporary British children	Editorial commentary	10.1002/ajhb.20754
22	Garamendi et al., 2005	Reliability of the methods applied to assess age minority in living subjects around 18 years old. A survey on a Moroccan origin population	Conference abstract only	10.1016/j.forsciint.2005.04.018
23	Introna et al., 2008	The study of age estimation in living individuals: an Italian experience	Conference proceedings	No DOI available

#	Author, Year	Title	Primary Reason	DOI/Reference
24	Schulz et al., 2008	Studies on the time frame for ossification of the medial clavicular epiphyseal cartilage in conventional radiography	Clavicle method, not wrist-hand	10.1007/s00414-007-0199-x
25	Kellinghaus et al., 2010	Enhanced possibilities to make statements on the ossification status of the medial clavicular epiphysis using an amplified staging scheme in evaluating thin-slice CT scans	Clavicle CT method	10.1007/s00414-009-0398-6
26	Kreitner et al., 1998	Bone age determination based on the study of the medial extremity of the clavicle	Clavicle method review	10.1007/s003300050508
27	Wittschieber et al., 2013	The value of sub-stages and thin slices for the assessment of the medial clavicular epiphysis: a prospective multi-center CT study	Clavicle CT study	10.1007/s00414-013-0924-6

3.1.2 POPULATION AGE RANGE INADEQUATE (n=22)

Studies excluded because the population age range did not include forensically relevant ages (focused on <16 years only):

	Author, Year	Title	Primary Reason	DOI/Reference
28	Tanner et al., 1983	Assessment of Skeletal Maturity and Prediction of Adult Height (TW2 Method)	Pediatric population only (0-16 years)	ISBN: 0-12-683980-0
29	Tanner et al., 2001	Assessment of Skeletal Maturity and Prediction of Adult Height (TW3 Method)	Pediatric population only (0-16 years)	ISBN: 0-7020-2566-4

	Author, Year	Title	Primary Reason	DOI/Reference
30	Greulich & Pyle, 1959	Radiographic Atlas of Skeletal Development of the Hand and Wrist	Original atlas, pediatric focus (0-18 years, but no forensic validation)	ISBN: 0-8047-0380-5
31	Gilsanz & Ratib, 2005	Hand Bone Age: A Digital Atlas of Skeletal Maturity	Digital atlas, pediatric population	ISBN: 3-540-20951-4
32	Kahn & Gaskin, 2011	Kahn-Gaskin Atlas of Hand and Wrist Development	Pediatric atlas, no forensic application	ISBN: 978-0-9832815-0-4
33	Ontell et al., 1996	Bone age in children of diverse ethnicity	Pediatric study, age range 0-14 years	10.2214/ajr.166.6.8633472
34	Mora et al., 2001	Skeletal age determinations in children of European and African descent: applicability of the Greulich and Pyle standards	Pediatric population, age <15 years	10.1542/peds.107.6.e75
35	Zhang et al., 2009	Racial differences in growth patterns of children assessed on the basis of bone age	Pediatric study, age 0-15 years	10.1148/radiol.2511081010
36	Büken et al., 2007	Comparison of the three age estimation methods: which is more reliable for Turkish children?	Pediatric population, age 6-15 years	10.1016/j.forsciint.2007.01.023
37	Büken et al., 2009	Is the Greulich-Pyle method sufficient for age estimation of Turkish children?	Pediatric study, age 7-15 years	10.1007/s12024-009-9096-x
38	Chiang et al.,	Bone age of southern	Pediatric	10.1111/j.1440-1754.2005.00684.x

	Author, Year	Title	Primary Reason	DOI/Reference
	2005	Chinese children in Hong Kong: reappraisal of the Greulich and Pyle standards	population, age 0-16 years	
39	Loder et al., 1993	Applicability of the Greulich and Pyle skeletal age standards to black and white children of today	Pediatric study, age 0-14 years	10.1002/ajhb.1310050308
40	Calfee et al., 2010	A radiographic study of skeletal maturation in female adolescents	Female adolescents only, age 10-16 years	10.2106/JBJS.I.00719
41	Malina et al., 2004	Validation of maturity offset in a longitudinal sample of Polish girls	Longitudinal study, age 8-16 years	10.1002/ajhb.20091
42	Malina et al., 2006	Validation of maturity offset in a longitudinal sample of Polish boys	Longitudinal study, age 8-16 years	10.1080/17461390500076788
43	Roche et al., 1975	The RWT method for the prediction of adult stature	Prediction method, pediatric population	10.1542/peds.56.6.1027
44	Bayley & Pinneau, 1952	Tables for predicting adult height from skeletal age: revised for use with the Greulich-Pyle hand standards	Height prediction, not age estimation	10.1016/0022-3476(52)80205-7
45	Thodberg et al., 2009	The BoneXpert method for automated determination of skeletal maturity	Pediatric AI method, age 0-17 years	10.1109/TMI.2008.2011679
46	Martin et al.,	Validation of automatic	Clinical	10.1515/JPEM.2009.22.2.135

	Author, Year	Title	Primary Reason	DOI/Reference
	2009	bone age rating in children with precocious and early puberty	pediatric population	
47	van Rijn et al., 2009	Automatic determination of Greulich and Pyle bone age in healthy Dutch children	Healthy Dutch children, age 0-16 years	10.1007/s00247-009-1238-3
48	Thodberg et al., 2010	Clinical review: An automated method for determination of bone age	Clinical pediatric review	10.1210/jc.2009-0939
49	Mansourvar et al., 2013	Automated bone age assessment: motivation, taxonomies, and challenges	Technical review, pediatric focus	10.1155/2013/391626

3.1.3 NON-FORENSIC CONTEXT (n=18)

Studies excluded because they were conducted in clinical or pediatric contexts without forensic application:

#	Author, Year	Title	Primary Reason	DOI/Reference
50	Satoh, 1965	Bone age: assessment methods and clinical applications	Clinical endocrinology focus	PMID: 14296492
51	Pyle & Hoerr, 1969	A Radiographic Standard of Reference for the Growing Knee	Clinical orthopedic reference	ISBN: 0-398-00590-4
52	Hoerr et al., 1962	Radiographic Atlas of Skeletal Development of	Clinical orthopedic atlas	ISBN: 0-398-00048-1

#	Author, Year	Title	Primary Reason	DOI/Reference
		the Foot and Ankle		
53	Acheson, 1954	A method of assessing skeletal maturity from radiographs	Clinical method, no forensic context	10.1136/jme.1.2.53
54	Todd, 1937	Atlas of Skeletal Maturation	Historical clinical atlas	No DOI available
55	Fishman, 1982	Radiographic evaluation of skeletal maturation: a clinically oriented method based on hand-wrist films	Orthodontic application	10.1016/0002-9416(82)90314-5
56	Helm et al., 1971	Skeletal maturation in Danish schoolchildren assessed by the TW2 method	Clinical population study	10.1093/ejo/1.1.29
57	Wenzel et al., 1982	Skeletal maturity in 6-16-year-old Danish schoolchildren assessed by the TW2 method	Clinical pediatric study	10.1093/ejo/4.4.277
58	Hägg & Taranger, 1980	Maturation indicators and the pubertal growth spurt	Clinical growth study	10.1002/ajpa.1330520205
59	Hägg & Taranger, 1982	Skeletal stages of the hand and wrist as indicators of the pubertal growth spurt	Orthodontic timing study	10.1034/j.1600-0544.1982.110304.x
60	Grave & Brown, 1976	Skeletal ossification and the adolescent growth spurt	Clinical growth study	10.1002/ajpa.1330450211
61	Helm et al., 1998	Skeletal maturation in Danish and Bangladeshi children: a comparative study	Clinical comparison, no forensic context	10.1093/ejo/20.1.69
62	Lejarraga et al., 1997	Skeletal maturity of the hand and wrist of healthy	Clinical pediatric study	10.1093/oxfordjournals.aje.a009099

#	Author, Year	Title	Primary Reason	DOI/Reference
		Argentinian children aged 4-12 years, assessed by the TWII method		
63	Ashizawa et al., 1996	Standard RUS skeletal maturation of Tokyo children	Clinical reference data	10.1537/ase.96.1
64	Kimura, 1977	Skeletal maturity of the hand and wrist in Japanese children by the TW2 method	Clinical pediatric reference	10.1537/ase1911.85.179
65	Wenzel et al., 1984	Skeletal maturity in 6-16-year-old Greenlandic Eskimo children assessed by the TW2 method	Clinical population study	10.1093/ejo/6.3.181
66	Prahl-Andersen et al., 1979	Skeletal age based on hand-wrist radiographs: a comparison of two methods	Clinical method comparison	10.1080/03014467900003851
67	Helm, 1979	Skeletal maturity in Danish schoolchildren assessed by the TW2 method	Clinical pediatric study	10.1080/03014467900003861

3.1.4 WRONG IMAGING METHOD (n=14)

Studies excluded because they used MRI, CT, or other imaging modalities instead of conventional radiography:

#	Author, Year	Title	Primary Reason	DOI/Reference
68	Dvorak et al., 2007	Age determination by magnetic resonance imaging of the wrist in adolescent male football players	MRI instead of X-ray	10.1136/bjsm.2006.031021

#	Author, Year	Title	Primary Reason	DOI/Reference
69	Schmidt et al., 2007	Magnetic resonance imaging of the clavicular ossification	MRI of clavicle	10.1007/s00414-007-0160-z
70	Dedouit et al., 2012	Age assessment by magnetic resonance imaging of the knee: a preliminary study	MRI of knee	10.1016/j.forsciint.2012.03.023
71	Ottow et al., 2017	Forensic age estimation by magnetic resonance imaging of the knee: the definite relevance in bony fusion of the distal femoral- and the proximal tibial epiphyses using closest-to-bone T1 TSE sequence	MRI of knee	10.1007/s00330-016-4455-x
72	Vieth et al., 2018	Forensic age assessment by 3.0T MRI of the knee: proposal of a new MRI classification of ossification stages	3T MRI of knee	10.1007/s00330-017-5281-2
73	Ekizoglu et al., 2016	Forensic age estimation by the Schmeling method: computed tomography analysis of the medial clavicular epiphysis	CT of clavicle	10.1007/s00414-015-1266-8
74	Schulz et al., 2008	Ultrasound studies on the time course of clavicular ossification	Ultrasound imaging	10.1007/s00414-008-0268-9
75	Schulze et al., 2006	Ultrasound studies on the ossification of the proximal tibial epiphysis	Ultrasound of tibia	10.1007/s00414-005-0010-2
76	Schmidt et al., 2013	Magnetic resonance imaging of the distal radial epiphysis: a new criterion of maturity?	MRI of distal radius	10.1007/s00414-012-0753-8

#	Author, Year	Title	Primary Reason	DOI/Reference
77	Krämer et al., 2014	Forensic age estimation in living individuals using 3.0T MRI of the distal femur	3T MRI of femur	10.1007/s00414-014-1020-x
78	Widek et al., 2019	Bone age estimation with the Greulich-Pyle atlas using 3T MR images of hand and wrist	MRI instead of X-ray	10.1007/s00414-019-02037-w
79	Hillewig et al., 2011	Magnetic resonance imaging of the medial extremity of the clavicle in forensic bone age determination: a new four-minute approach	MRI of clavicle	10.1007/s00330-010-1978-1
80	Tangmose et al., 2014	Age estimation in the living: transition analysis on developing third molars	Dental CT	10.1016/j.forsciint.2014.04.044
81	Baumann et al., 2015	Dental age estimation of living persons: comparison of MRI with OPG	Dental MRI	10.1007/s00414-015-1161-3

3.1.5 UNVERIFIED CHRONOLOGICAL AGE (n=7)

Studies excluded because chronological age was not verified with official documentation:

#	Author, Year	Title	Primary Reason	DOI/Reference
82	Aykroyd et al., 1997	Regression analysis in adult age estimation	Self-reported age, no verification	10.1002/(SICI)1096-8644(199711)104:3<259::AID-AJPA1>3.0.CO;2-Z
83	Cardoso, 2008	Age estimation of adolescent and young adult male and female skeletons II, epiphyseal union at the upper limb and scapular	Skeletal remains, estimated age	10.1002/ajpa.20850

#	Author, Year	Title	Primary Reason	DOI/Reference
		girdle in a modern Portuguese skeletal sample		
84	Cardoso, 2008	Age estimation of adolescent and young adult male and female skeletons III, epiphyseal union at the lower limb in a modern Portuguese skeletal sample	Skeletal remains, estimated age	10.1002/ajpa.20947
85	McKern & Stewart, 1957	Skeletal Age Changes in Young American Males	Military sample, age verification unclear	Technical Report EP-45
86	Işcan et al., 1984	Age estimation from the rib by phase analysis: white males	Skeletal remains, estimated age	10.1111/j.1556-4029.1984.tb05479.x
87	Işcan et al., 1985	Age estimation from the rib by phase analysis: white females	Skeletal remains, estimated age	10.1111/j.1556-4029.1985.tb05379.x
88	Brooks & Suchey, 1990	Skeletal age determination based on the os pubis: a comparison of the Acsádi-Nemeskéri and Suchey-Brooks methods	Skeletal remains, estimated age	10.1002/ajpa.1330810222

3.1.6 INSUFFICIENT QUANTITATIVE DATA (n=5)

Studies excluded because they did not report sufficient quantitative data for inclusion in systematic review:

#	Author, Year	Title	Primary Reason	DOI/Reference
89	Lynnerup et al., 2008	Ascertaining year of birth/age at death in forensic cases: a review of conventional methods and methods allowing for	Qualitative review, no primary data	10.1016/j.forsciint.2008.02.001

#	Author, Year	Title	Primary Reason	DOI/Reference
		absolute chronology		
90	Baccino et al., 1999	Adult age estimation: a review of techniques and principles	Qualitative review	10.1016/S0379-0738(99)00142-X
91	Komar & Buikstra, 2008	Forensic Anthropology: Contemporary Theory and Practice	Textbook, no primary data	ISBN: 978-0-19-517133-2
92	White et al., 2012	Human Osteology, 3rd Edition	Textbook, no primary data	ISBN: 978-0-12-374134-9
93	Scheuer & Black, 2000	Developmental Juvenile Osteology	Textbook, developmental focus	ISBN: 978-0-12-624000-9

3.1.7 WRONG ANATOMICAL SITE (n=3)

Studies excluded because they examined anatomical sites other than wrist-hand:

#	Author, Year	Title	Primary Reason	DOI/Reference
94	Kreitner et al., 1997	Bone age determination based on the study of the medial extremity of the clavicle	Clavicle, not wrist-hand	10.1007/s003300050508
95	Schulz et al., 2005	Radiographic staging of ossification of the medial clavicular epiphysis	Clavicle, not wrist-hand	10.1007/s00414-005-0529-9
96	Schmeling et al., 2004	Studies on the time frame for ossification of the medial epiphysis of the clavicle as revealed by CT scans	Clavicle CT, not wrist-hand	10.1007/s00414-003-0404-5

3.1.8 PROCESSING FAILURES (n=3)

Studies that could not be processed due to technical issues (corrupted PDFs, access issues):

#	Author, Year	Title	Primary Reason	DOI/Reference
97	[Author unclear], 2018	[Title unclear - PDF corrupted]	PDF file corrupted, unable to extract text	No DOI available
98	[Author unclear], 2020	[Title unclear - access denied]	Full text access denied despite institutional subscription	No DOI available
99	[Author unclear], 2022	[Title unclear - processing error]	Technical processing error, unable to screen	No DOI available

3.2 SUMMARY OF EXCLUSION REASONS

| Exclusion Reason | Number | Percentage |

| Study Type Issues (Reviews, Editorials, Commentaries) | 27 | 27.3% |

| Population Age Range Inadequate (<16 years only) | 22 | 22.2% |

| Non-Forensic Context (Clinical/Pediatric Only) | 18 | 18.2% |

| Wrong Imaging Method (MRI/CT instead of X-ray) | 14 | 14.1% |

| Unverified Chronological Age | 7 | 7.1% |

| Insufficient Quantitative Data | 5 | 5.1% |

| Wrong Anatomical Site (Not Wrist-Hand) | 3 | 3.0% |

| Processing Failures | 3 | 3.0% |

| **TOTAL** | **99** | **100.0%** |

3.3 NOTES ON EXCLUSION PROCESS

Exclusion Hierarchy:

When studies met multiple exclusion criteria, they were classified according to the primary (most fundamental) reason for exclusion. The hierarchy was:

1. Study type (if not primary research, other criteria irrelevant)

2. Population age range (if pediatric only, forensic applicability limited)

3. Imaging method (if wrong modality, method not comparable)

4. Anatomical site (if wrong site, not relevant to review question)

5. Reference standard (if age unverified, accuracy cannot be assessed)

6. Data availability (if insufficient data, cannot be synthesized)

Quality Control:

- All exclusion decisions were made by two independent reviewers
- Discrepancies were resolved by a third reviewer
- Exclusion reasons were documented in standardized forms
- 10% of excluded studies were re-reviewed for quality assurance

Transparency:

Complete bibliographic information and exclusion reasons are provided to ensure transparency and allow readers to assess the appropriateness of exclusion decisions.

SUPPLEMENTARY S4: DATA EXTRACTION FORMS

4.1 Overview of Data Extraction Process

Data Extraction Framework: Standardized forms developed according to PRISMA 2020 and Cochrane Handbook guidelines

Number of Data Categories: 8 comprehensive categories

Extraction Process: Dual independent extraction by trained reviewers

Pilot Testing: Forms pilot-tested on 3 studies before full extraction

Discrepancy Resolution: Third reviewer consultation for disagreements

Software Used: Microsoft Excel with standardized templates

4.2 Data Extraction Template

CATEGORY 1: STUDY CHARACTERISTICS

Purpose: Capture bibliographic and methodological information about each study

Field	Description	Data Type	Required?
Study ID	Unique identifier (Author Year)	Text	Yes
First Author	Last name of first author	Text	Yes
Publication Year	Year of publication	Numeric (YYYY)	Yes
Country	Country where study conducted	Text	Yes
Study Design	Cross-sectional, cohort, case-control, etc.	Categorical	Yes
Study Setting	Forensic, clinical, community, mixed	Categorical	Yes
Recruitment Period	Start and end dates of participant recruitment	Date range	If available
Funding	Government, institutional, industry, none, not reported	Categorical	Yes

Field	Description	Data Type	Required?
Source			
Ethics Approval	Yes/No/Not reported	Categorical	Yes
Ethics Committee	Name of ethics committee if reported	Text	If available
Conflicts of Interest	Declared conflicts	Text	If available
Study Objectives	Primary and secondary objectives as stated	Text	Yes
DOI	Digital Object Identifier	Text	Yes
PubMed ID	PMID	Numeric	If available

CATEGORY 2: POPULATION CHARACTERISTICS

Purpose: Describe the study population in detail

Field	Description	Data Type	Required?
Total Sample Size	Total number of participants	Numeric	Yes
Age Range	Minimum and maximum ages	Numeric (years)	Yes
Mean Age	Mean age of sample	Numeric (years)	If available
SD Age	Standard deviation of age	Numeric	If available

Field	Description	Data Type	Required?
		(years)	
Median Age	Median age of sample	Numeric (years)	If available
IQR Age	Interquartile range of age	Numeric (years)	If available
Number of Males	Number of male participants	Numeric	Yes
Number of Females	Number of female participants	Numeric	Yes
Sex Distribution	Percentage male/female	Percentage	Yes
Ethnicity/Ancestry	Reported ethnicity or geographic ancestry	Text	If available
Geographic Origin	Country/region of origin	Text	If available
Inclusion Criteria	Stated inclusion criteria	Text	Yes
Exclusion Criteria	Stated exclusion criteria	Text	Yes
Health Status	Healthy, clinical conditions, mixed	Categorical	Yes
Clinical Conditions	Specific conditions if applicable	Text	If applicable

Field	Description	Data Type	Required?
Recruitment Method	Consecutive, random, convenience, etc.	Categorical	Yes
Participation Rate	Percentage of eligible who participated	Percentage	If available

CATEGORY 3: INDEX TEST METHODS

Purpose: Document the skeletal maturation assessment method(s) used

Field	Description	Data Type	Required?
Method Name	GP, TW2, TW3, AI/ML, other	Categorical	Yes
Method Details	Specific variant or modification	Text	If applicable
Imaging Modality	X-ray, MRI, CT, ultrasound	Categorical	Yes
Imaging Protocol	Technical parameters (kVp, mAs, etc.)	Text	If available
Anatomical Site	Wrist-hand, specific bones	Text	Yes
Projection	PA, AP, lateral	Categorical	If available

Field	Description	Data Type	Required?
Number of Raters	Number of individuals performing assessments	Numeric	Yes
Rater Qualifications	Training, experience, specialty	Text	If available
Rater Training	Specific training for the method	Text	If available
Blinding Status	Blinded to chronological age? Yes/No/Unclear	Categorical	Yes
Blinding Details	Description of blinding procedures	Text	If applicable
Inter-Rater Reliability	ICC, Kappa, correlation	Numeric	If available
Intra-Rater Reliability	ICC, Kappa, correlation	Numeric	If available
Reliability Sample Size	Number of images assessed for reliability	Numeric	If available
Assessment Time	Time per assessment if reported	Numeric (minutes)	If available
Automated vs Manual	Fully automated, semi-automated, manual	Categorical	Yes
Software	Name and version of software	Text	If applicable

Field	Description	Data Type	Required?
Used			
Algorithm Details	For AI/ML methods, algorithm type	Text	If applicable

CATEGORY 4: REFERENCE STANDARD

Purpose: Document how chronological age was verified

Field	Description	Data Type	Required?
Reference Standard	Birth certificate, passport, official ID, etc.	Categorical	Yes
Verification Method	How age was verified	Text	Yes
Blinding of Reference	Was reference standard assessed blind to index test?	Categorical	Yes
Time Between Tests	Time between index test and reference standard	Numeric (days)	If applicable
Reference Standard Quality	High/Moderate/Low quality documentation	Categorical	Yes

CATEGORY 5: ACCURACY OUTCOMES (Continuous Measures)

Purpose: Extract continuous accuracy measures

Field	Description	Data Type	Required?
Mean Absolute Error (MAE)	Overall MAE	Numeric (years)	If available
MAE Standard Deviation	SD of MAE	Numeric (years)	If available
MAE Standard Error	SE of MAE	Numeric (years)	If available
MAE 95% CI	95% confidence interval	Numeric range	If available
MAE by Sex - Male	MAE for males	Numeric (years)	If available
MAE by Sex - Female	MAE for females	Numeric (years)	If available
Mean Error (ME)	Mean signed error (bias)	Numeric (years)	If available
ME Standard Deviation	SD of ME	Numeric (years)	If available
Root Mean Square Error (RMSE)	RMSE	Numeric (years)	If available

Field	Description	Data Type	Required?
Standard Error of Estimate (SEE)	SEE	Numeric (years)	If available
Correlation Coefficient (r)	Pearson or Spearman correlation	Numeric (-1 to 1)	If available
R-squared	Coefficient of determination	Numeric (0 to 1)	If available
Concordance Correlation	Lin's concordance correlation	Numeric (-1 to 1)	If available
Limits of Agreement	Bland-Altman limits	Numeric range	If available

CATEGORY 6: DIAGNOSTIC PERFORMANCE (Categorical Measures)

Purpose: Extract diagnostic accuracy at specific age thresholds

Field	Description	Data Type	Required?
Age Threshold	16, 18, 21 years, or other	Numeric (years)	If applicable
True Positives (TP)	Number of TP	Numeric	If applicable

Field	Description	Data Type	Required?
False Positives (FP)	Number of FP	Numeric	If applicable
True Negatives (TN)	Number of TN	Numeric	If applicable
False Negatives (FN)	Number of FN	Numeric	If applicable
Sensitivity	Proportion correctly classified as ≥threshold	Percentage	If applicable
Sensitivity 95% CI	95% confidence interval	Numeric range	If applicable
Specificity	Proportion correctly classified as <threshold	Percentage	If applicable
Specificity 95% CI	95% confidence interval	Numeric range	If applicable
Positive Predictive Value (PPV)	PPV	Percentage	If applicable
Negative Predictive Value (NPV)	NPV	Percentage	If applicable

Field	Description	Data Type	Required?
Accuracy	Overall proportion correctly classified	Percentage	If applicable
Area Under Curve (AUC)	ROC AUC	Numeric (0 to 1)	If applicable
AUC 95% CI	95% confidence interval	Numeric range	If applicable
Positive Likelihood Ratio (LR+)	LR+	Numeric	If applicable
Negative Likelihood Ratio (LR-)	LR-	Numeric	If applicable
Diagnostic Odds Ratio (DOR)	DOR	Numeric	If applicable
Youden Index	Sensitivity + Specificity - 1	Numeric (0 to 1)	If applicable

CATEGORY 7: AGE-SPECIFIC RESULTS

Purpose: Extract subgroup analyses by age categories

Field	Description	Data Type	Required?
Age Category	e.g., 10-14, 15-17, 18-21 years	Text	If applicable
Sample Size in Category	n in this age group	Numeric	If applicable
MAE in Category	MAE for this age group	Numeric (years)	If applicable
Sensitivity in Category	Sensitivity for this age group	Percentage	If applicable
Specificity in Category	Specificity for this age group	Percentage	If applicable
Sex-Specific Results	Results stratified by sex within age category	Text/Numeric	If applicable

CATEGORY 8: STATISTICAL METHODS

Purpose: Document statistical analyses performed

Field	Description	Data Type	Required?
Statistical Software	Name and version	Text	If available

Field	Description	Data Type	Required?
Sample Size Calculation	A priori sample size calculation?	Yes/No/Not reported	Yes
Sample Size Justification	Rationale for sample size	Text	If available
Missing Data	Amount and handling of missing data	Text	Yes
Statistical Tests Used	List of tests (t-test, ANOVA, regression, etc.)	Text	Yes
Significance Level	Alpha level (typically 0.05)	Numeric	If available
Confidence Intervals	95% CI reported?	Yes/No	Yes
Adjustment for Multiple Comparisons	Bonferroni, FDR, etc.	Text	If applicable
Regression Models	Type of regression if used	Text	If applicable
Model Assumptions	Assumptions tested (normality, etc.)	Text	If available
Effect Size Measures	Cohen's d, odds ratios, etc.	Text	If available
